# Taxonomic revision of the genus *Prionopelta* (Hymenoptera, Formicidae) in the Malagasy region

**DOI:** 10.3897/zookeys.507.9303

**Published:** 2015-06-09

**Authors:** Rick Overson, Brian L. Fisher

**Affiliations:** 1 Chicago Botanic Garden, 1000 Lake Cook Road, Glencoe, IL 60022, U.S.A.; 2 Entomology, California Academy of Sciences, 55 Music Concourse Drive, San Francisco, CA 94118, U.S.A.

**Keywords:** Afrotropical region, Madagascar, equatorial rainforest, taxonomy, Amblyoponinae, *Prionopelta*

## Abstract

In this study we revise the taxonomy of the genus *Prionopelta* for the Malagasy region, treating seven species, six of which are newly described (*Prionopelta laurae* **sp. n.**, *Prionopelta seychelles* **sp. n.**, *Prionopelta subtilis* **sp. n.**, *Prionopelta talos* **sp. n.**, *Prionopelta vampira* **sp. n.**, *Prionopelta xerosilva* **sp. n.**), and one redescribed (*Prionopelta descarpentriesi* Santschi). One species, *Prionopelta seychelles*, is restricted to Seychelles, while the six remaining species treated are endemic to Madagascar.

## Introduction

The genus *Prionopelta* Mayr, 1866 contains 21 species, including six that are newly described here. In his species-level treatment of the genus, Brown (1960) recognized ten species scattered throughout the Old and New World tropics, and later returned *Prionopelta marthae* to the genus after an erroneous removal based on mislabeled specimens (Brown 1965). Subsequently, Terron described two new species from the Afrotropics (1974), and Shattuck described two new Indo-Pacific species and clarified diagnoses and geographic distributions for *Prionopelta kraepelini* and *Prionopelta opaca* (2008). Total species counts to date are thus: five from the New World tropics (*Prionopelta punctulata* Mayr, *Prionopelta antillana* Forel, *Prionopelta marthae* Forel, *Prionopelta modesta* Forel, and *Prionopelta amabilis* Borgmeier), six from the Indo-Pacific region: (*Prionopelta majuscula* Emery, *Prionopelta opaca* Emery, *Prionopelta kraepelini* Forel, *Prionopelta brocha* Wilson, *Prionopelta media* Shattuck, and *Prionopelta robynmae* Shattuck), three from the tropics of Africa (*Prionopelta amieti* Terron, *Prionopelta humicola* Terron, *Prionopelta aethiopica* Arnold), and seven from the Malagasy region (*Prionopelta descarpentriesi* Santschi, *Prionopelta laurae* **sp. n.**, *Prionopelta seychelles* **sp. n.**, *Prionopelta subtilis* **sp. n.**, *Prionopelta talos* **sp. n.**, *Prionopelta vampira* **sp. n.**, *Prionopelta xerosilva* **sp. n.**). Interestingly, *Prionopelta* is thus one of four genera, along with *Mystrium*, *Euponera*, and *Leptogenys*, where described species numbers from the Malagasy region are greater than those from the Afrotropics.

## Material and methods

The present contribution includes all specimens of *Prionopelta* collected from the arthropod survey project conducted in Madagascar by B.L. Fisher and the members of the Madagascar Biodiversity Center from 1992 through 2013 (Fisher 2005). This revision also integrates specimens from the Philip S. Ward Collection at the University of California Davis, CA, U.S.A. (PSWC). Observations were carried out with a Leica MZ125 microscope. Digital color montage images were created using a JVC KY-F75 digital camera and Syncroscopy Auto-Montage software (version 5.0), or a Leica DFC 425 camera in combination with the Leica Application Suite software (version 3.8). All images presented here are available online at AntWeb (http://www.antweb.org). Distribution maps for each species were produced with the software R (R Core Team 2015). Measurements were performed using a Mitutoyo digital, dual-axis stage micrometer. Measurements were recorded to the thousandth of a millimeter, but values presented are rounded to the nearest hundredth of a millimeter. For each species, measurements are presented as minimums and maximums with means following in parentheses. The following measurements and indices are reported, largely following Shattuck (2008):

HW Head width: maximum head width in full-face (dorsal) view.

HL Head length: maximum head length in full-face (dorsal) view, measured from the anteriormost point of the clypeal margin to the posteriormost point of the head proper.

SL Scape length: length of the scape (first antennal segment) excluding the basal neck and condyle.

WL Weber’s length: diagonal length of mesosoma in lateral view from the posteroventral margin of propodeal lobe to the anteriormost point of pronotal slope, excluding the neck.

PetL Petiole length: midline length of the petiolar node (excluding the anterior peduncle) in dorsal view.

PetW Petiole width: width of the petiolar node in dorsal view.

T1W First gastral segment width: width of first gastral (third abdominal) tergite in dorsal view.

CI Cephalic index: HW / HL × 100

SI Scape index: SL / HW × 100

PI Petiolar index: PetW / PetL × 100

### Abbreviations of depositories

Collection abbreviations follow Bolton (1980) and Evenhuis (2009). The material upon which this study is based is located, or deposited at the following institutions:

BMNH The Natural History Museum (British Museum, Natural History), London, U.K.

CASC California Academy of Sciences, San Francisco, California, U.S.A.

MCZC Museum of Comparative Zoology, Cambridge, Mass. U.S.A.

MHNG Muséum d’Histoire Naturelle, Geneva, Switzerland

NHMB Naturhistorisches Museum, Basel, Switzerland

PBZT Parc Botanique et Zoologique de Tsimbazaza, Antananarivo, Madagascar

PSWC Philip S. Ward Collection at the University of California Davis, CA, U.S.A.

### The role of sculpture in identifying species of Malagasy *Prionopelta*

Sculpture of the head and dorsum of the mesosoma is of high diagnostic value in identifying Malagasy *Prionopelta*. In describing sculpture we use the same terminology as Harris (1979) and Shattuck (2008). We use the term “foveae” to refer to the shallow, circular, flat-bottomed depressions present on the integument, and the terms “punctations” or “punctures” to mean minute, point-like depressions in the surface of the integument that appear as tiny pinpricks even under high magnification. All Malagasy *Prionopelta* have foveae that vary in both size and density on both head and mesosoma, and most species also have punctations on the dorsum of the mesosoma. Additionally, all taxa possess a band devoid of foveae on the head that runs medially and longitudinally. This area, extending from just posterior to the antennal sockets to near the posterior margin of the head, varies in size and shape among species. Throughout the text, this area is excluded from consideration when statements are made concerning the density and extent of sculpture on the head. In some individuals a linear, scarlike, coronal suture is present in this region running longitudinally along the dorsum of the head.

**Figure 1.**
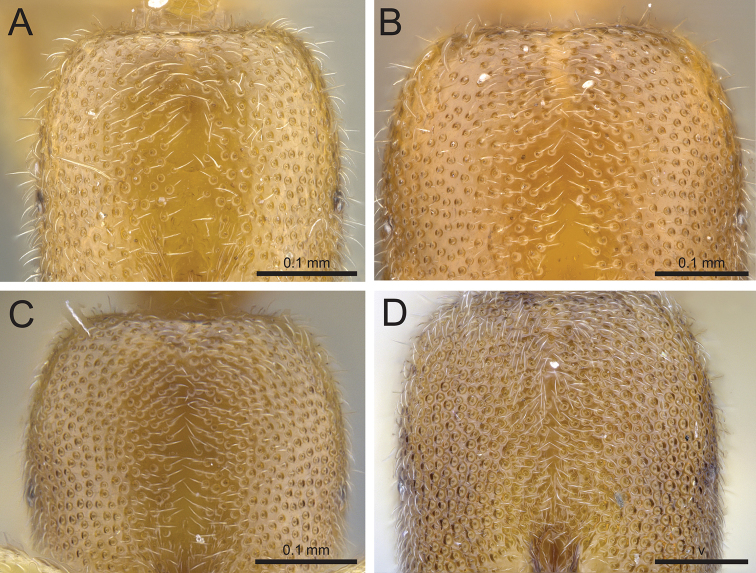
Head in full-face view. **A** *Prionopelta xerosilva* (CASENT0157254) **B** *Prionopelta vampira* (CASENT0461978) **C** *Prionopelta descarpentriesi* “morphotype-A” (CASENT0034837) **D** *Prionopelta descarpentriesi* “morphotype-C” (CASENT0191895).

When identifying Malagasy *Prionopelta*, it is helpful to recognize three qualitative conditions concerning the arrangement of foveae on the head. The first condition occurs when foveae are relatively equally spaced from one another at a distance that is greater than the diameter of an individual fovea, with smooth, shining integument present between foveae (Fig. 1A, B). The next condition is one of variable foveal placement, with some foveae as described above but others directly adjacent to one another (Fig. 1C). At low magnification, these adjacent foveae appear to touch directly, whereas under high magnification they appear very close together (much closer together than the diameter of an individual fovea), causing the area between each fovea to form a distinct raised margin that rises above what would otherwise be the smooth surface of the integument. There is a general tendency in both of the above-described conditions for cephalic foveae to increase in density moving laterally to medially in full-face view. In the final of these three general conditions, foveae are placed extremely densely on the head so that virtually no shining integument is present between foveae (except for the median cephalic band mentioned above). In this condition, foveae appear as a dense system of directly adjacent pits with only netlike boundaries of thin, raised margins separating individual fovea (Fig. 1D).

## Taxonomy

### *Prionopelta* Mayr, 1866

*Prionopelta* can be recognized from other Malagasy genera through the following combination of characters: petiole which lacks a posterior face due to its broad attachment to the gaster; elongate, subtriangular mandibles with three teeth positioned distally on a distinct mandibular face; apical tooth the longest, and second tooth the shortest with a mid-length third tooth; antennal segments variable (either 9 or 12 for Malagasy species) but always with a four-segmented club; body overall never more than 3 mm in length.

### Synopsis of Malagasy *Prionopelta*

*Prionopelta descarpentriesi* Santschi, 1924

*Prionopelta laurae* **sp. n.**

*Prionopelta seychelles* **sp. n.**

*Prionopelta subtilis* **sp. n.**

*Prionopelta talos* **sp. n.**

*Prionopelta vampira* **sp. n.**

*Prionopelta xerosilva* **sp. n.**

### Identification key for Malagasy *Prionopelta* species (workers)

**Table:** 

1	Nine antennal segments present, palest and smallest of the Malagasy *Prionopelta* (HL < 0.4 mm and HW < 0.3 mm); entire body pale yellow (Fig. 7)	***Prionopelta laurae***
–	Twelve antennal segments present; not as small as above (HL > 0.4 mm and HW > 0.3 mm); varying in size and color	**2**
2	Cephalic foveae widely and evenly spaced such that they are only extremely rarely adjacent to one another; all cephalic foveae appear as if scooped out of a flat, shining surface, and completely lack raised margins at their perimeter (Fig. 1A, B)	**3**
–	Cephalic foveae of variable spacing but never as sparse as above; at a minimum, head with several clusters of two or more foveae which are directly adjacent in full-face view (Fig. 1C) and often with many to most foveae on the head directly adjacent to one another (Fig. 1D); integument between adjacent foveae appears to bulge, and when foveae are densely placed, these bulged areas form a network of raised margins (Fig. 1D)	**4**
3	Metanotal suture absent in dorsal view, in its place a smooth surface with no clear boundary dividing mesonotum and propodeum; posterior margin of the propodeum convex and crescent-shaped in dorsal view (Fig. 2A); lamellae of the posterior propodeum present; apical tooth of the mandible extremely long (Fig. 2C)	***Prionopelta vampira***
–	Metanotal suture present; posterior margin of the propodeum relatively straight in dorsal view (Fig. 2B); lamellae absent; apical tooth intermediate in length (Fig. 2D); known only from tropical dry forests of western Madagascar	***Prionopelta xerosilva***
4	Vast majority of cephalic foveae densely positioned so they are directly adjacent to one another; vein-like ridges present running between foveae producing the appearance of a netlike pattern across the entire head (Fig. 3A, B, C, D); areas of shining integument devoid of foveae are present only at the extreme posterolateral corners of the head	**5**
–	Cephalic foveae variable in their spacing, but always with some foveae directly adjacent and others isolated from one another; usually with the frequency of adjacent foveae increasing laterally to medially (Fig. 1C)	**7**
5	Coronal suture present on head which appears as a uniformly thin, linear scar that swells above the surrounding integument under high magnification (Fig. 3A, B); sculpture on pronotum weak, consisting of shallow foveae which are much larger and more widely spaced than those on the head, and are interspersed with minute punctures.	***Prionopelta subtilis***
–	Coronal suture absent on head; median cephalic band wider and more irregular, never uniformly swelling above the surface of the surrounding integument as a suture, appearing instead as a shining area devoid of foveae (Fig. 3C); if band is narrow (Fig. 3D), then surface of the pronotum always covered in large, deep, regularly-spaced foveae, similar in size to those on the head	**6**
6	Cephalic foveae small and densely positioned so that the vast majority are directly adjacent to one another with swollen ridges between; median cephalic band devoid of foveae is wide, usually wider at its base and narrowing posteriorly (Fig. 3C); foveae of the pronotum much larger than that of the head, ranging from shallow to deep, and interspersed with punctures; known only from Seychelles	***Prionopelta seychelles***
–	Cephalic foveae large, deep, and densely placed, joining to form a network of tall, jagged ridges; median cephalic band lacking foveae is usually thin and narrow, but its boundaries are irregular and jagged (Fig. 3D); pronotum lacking punctures and possessing deep, regularly-spaced foveae which are similar-sized to those on the head	***Prionopelta descarpentriesi*** (in part)
7	Distinctly tricolored body with dark brown, uniformly colored head, lighter brown body, and pale yellow legs (Fig. 10B); large, globular eyes which project spherically from the head; at high magnification, eyes composed of several overlapping globular sections delineated by sutures (Fig. 4A, D); known only from the Anjanaharibe-Sud Reserve of Madagascar	***Prionopelta talos***
–	Not distinctly tricolored as above; some individuals possess dark brown on head but always at least with lighter brown present at the posterolateral corners of the head which resembles the color of the mesosoma (Fig. 5A, B); eyes ranging in size from almost absent (Fig. 4C, F) to dark, irregular circles that are almost flush with the surrounding integument (Fig. 4B, E); eyes never globular and never composed of several visible globular sections	***Prionopelta descarpentriesi*** (in part)

**Figure 2.**
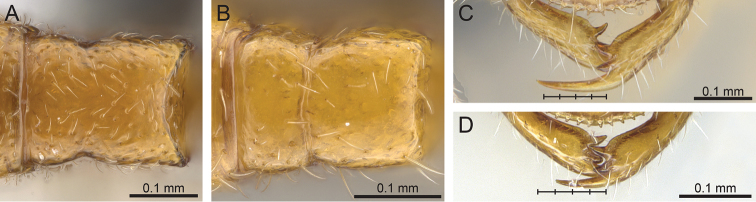
**A**, **B** Mesonotum and propodeum in dorsal view. **A** *Prionopelta vampira* (CASENT0461978) **B** *Prionopelta xerosilva* (CASENT0157254) **C** mandibles of *Prionopelta vampira*, demonstrating that apical tooth is greater than four times the length of third tooth (CASENT0461978) **D** mandibles of *Prionopelta xerosilva*, demonstrating that apical tooth is less than four times the length of third tooth (CASENT0157254).

**Figure 3.**
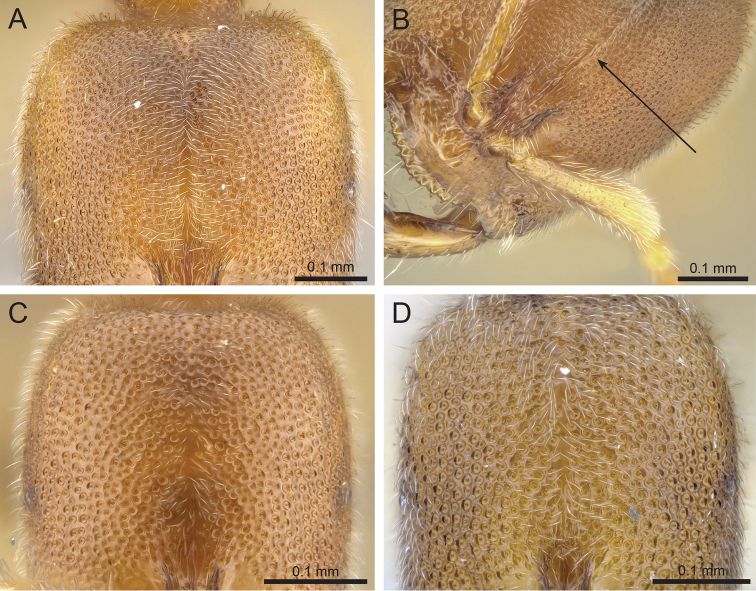
Head in full-face view. **A** *Prionopelta subtilis* (CASENT0033641) **B** *Prionopelta subtilis* (CASENT0151601) specimen rotated to demonstrate raised coronal suture **C** *Prionopelta seychelles* (CASENT0161311) **D** *Prionopelta descarpentriesi* “morphotype-C” (CASENT0191895).

**Figure 4.**
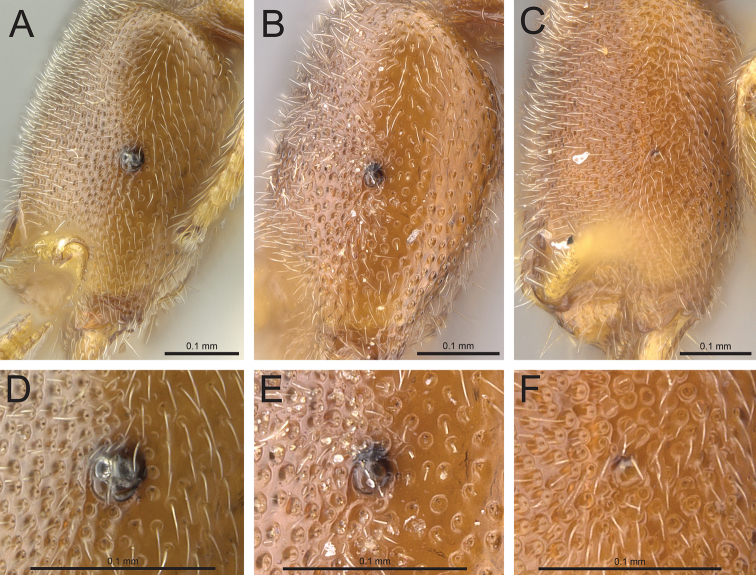
**A, B, C** Head in profile. **A** *Prionopelta talos* (CASENT0339229) **B** *Prionopelta descarpentriesi* “morphotype-A” (CASENT0168970) (photo: Michele Esposito 2014) **C** *Prionopelta descarpentriesi* “morphotype-C” (CASENT0042668) (photo: Michele Esposito 2014) **D, E, F** close-up of eye. **D** *Prionopelta talos* (CASENT0339229) **E** *Prionopelta descarpentriesi* (CASENT0168970) (Michele Esposito 2014) **F** *Prionopelta descarpentriesi* (CASENT0042668) (Michele Esposito 2014).

**Figure 5.**
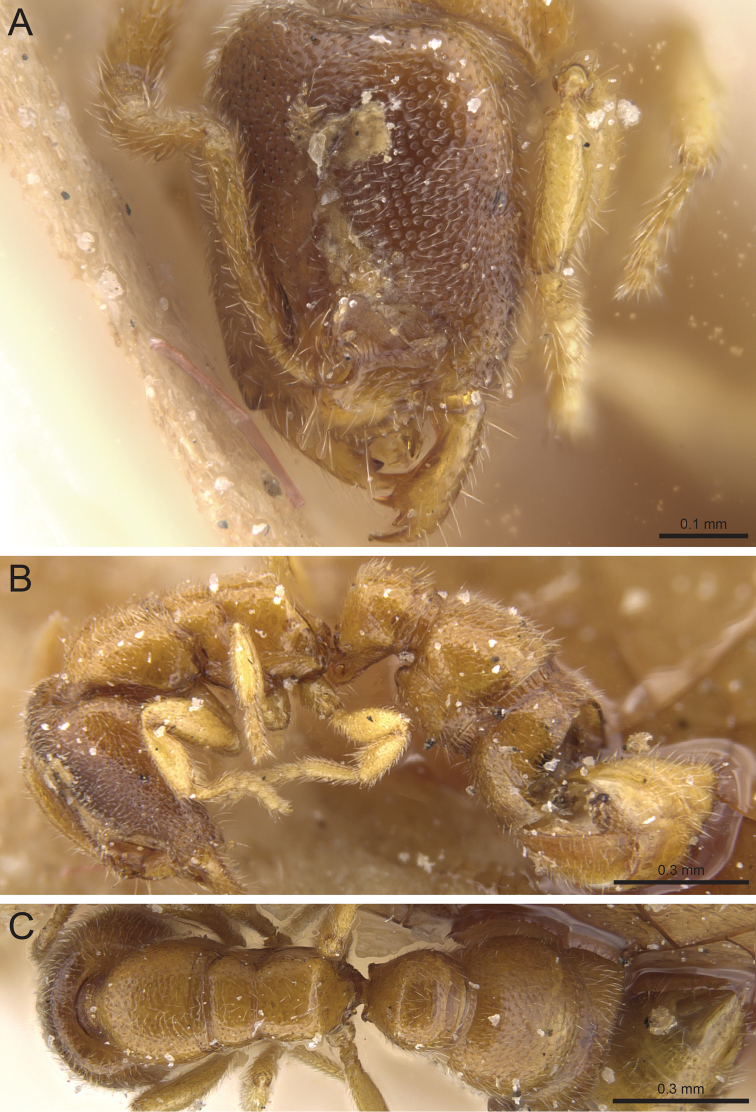
*Prionopelta descarpentriesi* lectotype worker (CASENT0101547). **A** Head in full-face view **B** Body in profile **C** Body in dorsal view.

### Prionopelta descarpentriesi

Taxon classificationPlantaeHymenopteraFormicidae

Santschi

**Figure 6.**
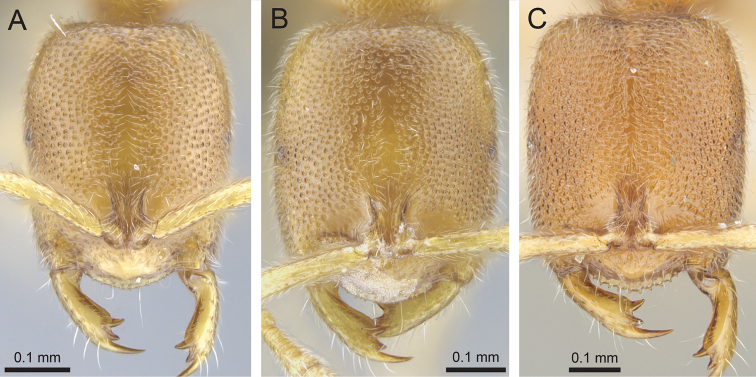
**A, B, C** Head in full-face view displaying variation in *Prionopelta descarpentriesi* for cephalic sculpture. **A** (CASENT0034837) “morphotype-A” **B** (CASENT0480792) “morphotype-B” **C** (CASENT0191895) “morphotype-C”.

Figs 1C, D , 3D , 4B, C, E, F , 5 , 6

Prionopelta descarpentriesi Santschi, 1924b:195.

#### Type material.

**Lectotype**, pinned worker, CASENT0101548 [designated here], MADAGASCAR, Ikelivia, 30.ix.1923 (*Descarpentries*) (NHMB) [examined]. **Paralectotypes**, one pinned worker CASENT0101547 with same data as lectotype (NHMB) [examined].

#### Diagnosis.

*Prionopelta descarpentriesi* can be identified by the following combination of characters: twelve antennal segments; median cephalic band lacking a thin suture that is swollen above the surrounding integument; placement of cephalic foveae ranging from sparse to dense, but always at minimum, with at least several clusters of foveae directly adjacent to one another (if nowhere else, then medially in full-face view); if all foveae on the head are directly adjacent so that no flat, shining space is present between foveae, then foveae are large and accompanied by pronotal sculpture which is characterized as being both similar in size to that on the head and not consisting of smaller foveae or punctures; eye appearing as either an asymmetrical dark patch which appears to be a stain in the cuticle that is flush with its surrounding integument, or a single, slightly rounded glob with no definable subunits.

#### Worker measurements

**(N=25).** HL 0.42–0.53 (0.48); HW 0.32–0.43 (0.38); SL 0.22–0.3 (0.26); WL 0.46–0.6 (0.52); PetL 0.11–0.2 (0.16); PetW 0.17–0.26 (0.22); T1W 0.29–0.37 (0.33); CI 74.64–85.1 (79.6); PI 118.23–173.29 (141.94); SI 61.46–72.38 (67.31).

#### Worker description.

Posterior margin of the head straight to weakly concave in full-face view; spacing of cephalic foveae highly variable, ranging from individuals with dense, directly adjacent foveae covering the entire head (known only from far eastern and northern Madagascar, see morphotype descriptions below), to individuals with foveae more widely spaced so that shining areas are visible between; median cephalic band devoid of foveae ranging from wide to extremely narrow but never appearing as a linear suture that is uniformly swollen above the level of the surrounding integument; apical tooth intermediate in length; evenly-space pronotal foveae range from shallow to deep; shallow foveae present on mesonotum and propodeum.

#### Distribution and ecology.

This widespread species has been collected from leaf litter from 10–1860 meters of elevation. While found most commonly in rainforest and montane rainforest, it has also been collected in Uapaca woodland, littoral rainforest, and tropical dry forest (Fig. 13). *Prionopelta descarpentriesi* has been collected in forest litter, under moss, rocks, and logs, as well as inside rotten logs and underground in soil.

#### Taxonomic notes.

Three generalized morphotypes can be distinguished within this taxon; these vary in density of cephalic foveae and other co-occurring traits (Fig. 6). In morphotype A, the majority of foveae are equidistantly spaced and separated by a span of shining integument of around one foveal diameter (Figs 1C, 5A, 6A). These foveae appear cleanly scooped from the surface of the integument and largely lack raised margins. Morphotype B, which is intermediate between A and C, has denser cephalic foveae covering almost the entire head, however foveae are smaller and more delicate than those of morphotype C and raised ridges between foveae are less pronounced (Fig. 6B). Pronotal sculpture in morphotype B usually consists of several sizes of foveae along with some punctures. Morphotype C possesses large, dense cephalic foveae that cover the entire head, accompanied by a pronounced network of raised, jagged ridges between foveae (Figs 1D, 3D, 4C, 6C). Morphotype C additionally has large, deep, and evenly spaced pronotal foveae that are similar in size to those on the head, and this morphotype lacks smaller foveae or punctures on the pronotum. Width of the median cephalic band devoid of foveae is widest in morphotype A and narrowest in morphotype C.

*Prionopelta descarpentriesi* is much more morphologically variable than the other species treated in this revision and very possibly represents a species complex. This could explain why *Prionopelta descarpentriesi* is abundant, geographically widespread, and morphologically variable. Morphotype C, which is restricted to a band bordering the coast of eastern and northern Madagascar (Fig. 14), is recognizably distinct from the majority of individuals from interior populations of the species. At several localities where morphotype C is present, individuals from morphotype A and B are also present with little to no evidence of character blending between morphotypes: Galako, Makirovana, Morojejy Nature Reserve, and Sahafina. However, at other locales such as Ambohijanahary, Montagne d’Ambre, and Vohemar, a bewildering array of intermediate forms have been collected, blurring the lines between the three morphotypes. Collecting nest series from the aforementioned locations to determine whether this population-level variation is intra- or intercolonial is an important first step in ultimately understanding how morphological variation is partioned in this taxon as currently dilineated.

Under the above scheme, the lectotype and paralectotype of *Prionopelta descarpentriesi* would be considered morphotype A. Jules Descarpentries (1881–1927) collected the types for this species on 30 September 1923 and the collection locale was subsequently noted as “Ikelivia”, making it difficult to determine a more precise location based on modern place names. Historical records indicate that Descarpentries resided in Tulear, and worked as a topographic surveyor and entomologist. According to records, he was active around Tsaratanana and Fianarantsoa (specifically Andringitra) and often traveled with H. Perrier de la Bâthie. Given that morphotype A is reasonably widespread across Madagascar, including Tsaratanana and Andringitra, it is possible that the type specimens from “Ikelivia” were collected in either of these localities. To further complicate matters, most of Descarpentries’s specimens, which were destined to the Paris Museum, were sold on the side by a member of the staff. Before the specimens were sold, the labels were changed to hide their true ordinance (Jeannel 1951, (Anonymous 1970)).

#### Non-type material.

MADAGASCAR: Antananarivo, Ankalalahana, 19.0066°S, 47.1122°E, 1375 m, Uapaca woodland, 29.iii.2011 (*B.L.Fisher et al.*); Antananarivo, Forêt de galerie, Andranorovitra, 24.0 km NNE Ankazobe, 18.1124°S, 47.1976°E, 1491 m, disturbed gallery montane forest, 2.vi.2008 (*B.L.Fisher et al.*); Antananarivo, Navoatra I Non Protected Area, 7.64 km NW Arivonimamo, 18.9781°S, 47.1193°E, 1373 m, Uapaca woodland, 6.v.2010 (*Andrianjaka Ravelomanana*); Antananarivo, Réserve Naturelle Sohisika, Sohisika 24.6 km NNE Ankazobe, 18.1032°S, 47.1869°E, 1464 m, gallery montane forest, 1.vi.2008 (*B.L.Fisher et al.*); Antananarivo, Réserve Spéciale d’Ambohitantely, 18.2244°S, 47.2774°E, 1490 m, montane forest, 9.iii.2012 (*B.L.Fisher et al.*); Antananarivo, Réserve Spéciale d’Ambohitantely, Forêt d’Ambohitantely, 20.9 km 72° NE d’Ankazobe, 18.2253°S, 47.2868°E, 1410 m, montane rainforest, 17.iv.2001 (*Fisher, Griswold et al.*); Antananarivo, Réserve Spéciale d’Ambohitantely, Forêt d’Ambohitantely, Jardin Botanique, 24.1 km 59° NE d’Ankazobe, 18.1714°S, 47.2818°E, 1620 m, montane rainforest, 17.iv.2001 (*Fisher, Griswold et al.*); Antananarivo, Tsimbazaza, 18.928°S, 47.527°E, 1300 m, park/garden, 16.xii.2006 (*B.L.Fisher et al.*); Antsiranana, 6.5 km SSW Befingotra, Rés. Anjanaharibe-Sud, 14.75°S, 49.5°E, 875 m, rainforest, 19.x.1994 (*B.L.Fisher*); Antsiranana, 9.2 km WSW Befingotra, Rés. Anjanaharibe-Sud, 14.75°S, 49.4667°E, 1280 m, montane rainforest, 5.xi.1994 (*B.L.Fisher*); Antsiranana, Ambondrobe, 41.1 km 175° Vohemar, 13.7153°S, 50.1017°E, 10 m, littoral rainforest, 29.xi.2004 (*B.L.Fisher*); Antsiranana, Ampasindava, Andranomatavy Forest, 13.669°S, 47.9877°E, 149 m, disturbed dry forest, 6.x.2013 (*B.L.Fisher et al.*); Antsiranana, Ampasindava, Andranomatavy Forest, 13.663°S, 47.9794°E, 543 m, disturbed dry forest, 6.x.2013 (*B.L.Fisher et al.*); Antsiranana, Binara Forest, 13.2621°S, 49.6067°E, 559 m, degraded rainforest, 18.x.2013 (*B.L.Fisher et al.*); Antsiranana, Binara Forest, 13.2621°S, 49.605°E, 692 m, rainforest, 18.x.2013 (*B.L.Fisher et al.*); Antsiranana, Binara Forest, 13.2639°S, 49.5992°E, 1065 m, rainforest, 18.x.2013 (*B.L.Fisher et al.*); Antsiranana, Forêt Ambanitaza, 26.1 km 347° Antalaha, 14.6793°S, 50.1837°E, 240 m, rainforest, 26.xi.2004 (*B.L.Fisher*); Antsiranana, Forêt d’ Andavakoera, 21.4 km 75° ENE Ambilobe; 4.6 km 356° N Betsiaka, 13.1183°S, 49.23°E, 425 m, rainforest, 15.xii.2003 (*B.L.Fisher*); Antsiranana, Forêt d’ Antsahabe, 11.4 km 275° W Daraina, 13.2117°S, 49.5567°E, 550 m, tropical dry forest, 12.xii.2003 (*B.L.Fisher*); Antsiranana, Forêt d’Ampondrabe, 26.3 km 10° NNE Daraina, 12.97°S, 49.7°E, 175 m, tropical dry forest, 10.xii.2003 (*B.L.Fisher*); Antsiranana, Forêt de Bekaraoka, 6.8 km 60° ENE Daraina, 13.1667°S, 49.71°E, 150 m, tropical dry forest, 7.xii.2003 (*B.L.Fisher*); Antsiranana, Forêt de Binara, 7.5 km 230° SW Daraina, 13.255°S, 49.6167°E, 375 m, tropical dry forest, 1.xii.2003 (*B.L.Fisher*); Antsiranana, Forêt de Binara, 9.1 km 233° SW Daraina, 13.2633°S, 49.6033°E, 800 m, rainforest, 3.xii.2003 (*B.L.Fisher*); Antsiranana, Forêt de Binara, 9.4 km 235° SW Daraina, 13.2633°S, 49.6°E, 1100 m, montane rainforest, 5.xii.2003 (*B.L.Fisher*); Antsiranana, Galoko chain, Mont Galoko, 13.5936°S, 48.7316°E, 1100 m, montane forest, 22.ii.2013 (*B.L.Fisher et al.*); Antsiranana, Galoko chain, Mont Galoko, 13.5888°S, 48.7286°E, 980 m, montane forest, 22.ii.2013 (*B.L.Fisher et al.*); Antsiranana, Galoko chain, Mont Galoko, 13.5849°S, 48.7182°E, 520 m, rainforest, 16.ii.2013 (*B.L.Fisher et al.*); Antsiranana, Galoko chain, Mont Kalabenono, 13.6418°S, 48.6728°E, 643 m, rainforest, 10.x.2013 (*B.L.Fisher et al.*); Antsiranana, Galoko chain, Mont Kalabenono, 13.64°S, 48.6737°E, 498 m, rainforest, 10.x.2013 (*B.L.Fisher et al.*); Antsiranana, Galoko chain, Mont Kalabenono, 13.6461°S, 48.6773°E, 937 m, rainforest, 10.x.2013 (*B.L.Fisher et al.*); Antsiranana, Makirovana forest, 14.1604°S, 49.9522°E, 550 m, rainforest, 1.v.2011 (*B.L.Fisher et al.*); Antsiranana, Makirovana forest, 14.1651°S, 49.9477°E, 900 m, montane rainforest, 30.iv.2011 (*B.L.Fisher et al.*); Antsiranana, Makirovana forest, 14.1667°S, 49.95°E, 715 m, rainforest, 1.v.2011 (*B.L.Fisher et al.*); Antsiranana, Makirovana forest, 14.1707°S, 49.9541°E, 415 m, rainforest, 28.iv.2011 (*B.L.Fisher et al.*); Antsiranana, Parc National de Marojejy, Antranohofa, 26.6 km 31° NNE Andapa, 10.7 km 318° NW Manantenina, 14.4433°S, 49.7433°E, 1325 m, montane rainforest, 18.xi.2003 (*B.L.Fisher*); Antsiranana, Parc National de Marojejy, Manantenina River, 27.6 km 35° NE Andapa, 9.6 km 327° NNW Manantenina, 14.435°S, 49.76°E, 775 m, rainforest, 15.xi.2003 (*B.L.Fisher et al.*); Antsiranana, Parc National de Marojejy, Manantenina River, 28.0 km 38° NE Andapa, 8.2 km 333° NNW Manantenina, 14.4367°S, 49.775°E, 450 m, rainforest, 12.xi.2003 (*B.L.Fisher et al.*); Antsiranana, Parc National Montagne d’Ambre, 12.2 km 211° SSW Joffreville, 12.5964°S, 49.1595°E, 1300 m, montane rainforest, 2.ii.2001 (*Fisher, Griswold et al.*); Antsiranana, Parc National Montagne d’Ambre, 3.6 km 235° SW Joffreville, 12.5344°S, 49.1795°E, 925 m, montane rainforest, 20.i.2001 (*Fisher, Griswold et al.*); Antsiranana, Prov.Antsiranana R.S. Manongarivo 17.3 km 218° SW Antanambao, 14.0217°S, 48.4183°E, 1580 m, montane rainforest, 27.x.1998 (*B.L.Fisher*); Antsiranana, R.S. Manongarivo, 10.8 km 229° SW Antanambao, 13.9617°S, 48.4333°E, 400 m, rainforest, 8.xi.1998 (*B.L.Fisher*); Antsiranana, R.S. Manongarivo, 12.8 km 228° SW Antanambao, 13.9767°S, 48.4233°E, 780 m, rainforest, 11.x.1998 (*B.L.Fisher*); Antsiranana, R.S. Manongarivo, 14.5 km 220° SW Antanambao, 13.9983°S, 48.4283°E, 1175 m, montane rainforest, 20.x.1998 (*B.L.Fisher*); Antsiranana, R.S. Manongarivo, 20.4 km 219° SW Antanambao, 14.0467°S, 48.4017°E, 1860 m, montane rainforest, 3.xi.1998 (*B.L.Fisher*); Fianarantsoa, 28 km. SSW Ambositra, Ankazomivady, 20.775°S, 47.1683°E, 1670 m, grassland, 11.i.1998 (*B.L.Fisher*); Fianarantsoa, 3 km W Ranomafana, nr. Ifandiana, 21.25°S, 47.4167°E, 950 m, forest, 27.iv.1989 (*P.S.Ward*); Fianarantsoa, 43 km S Ambalavao, Rés. Andringitra, 22.2333°S, 47°E, 825 m, rainforest, 5.x.1993 (*B.L.Fisher*); Fianarantsoa, 45 km S. Ambalavao, 22.2167°S, 47.0167°E, 785 m, rainforest, 25.ix.1993 (*B.L.Fisher*); Fianarantsoa, 7.6 km 122º Kianjavato, Forêt Classée Vatovavy, 21.4°S, 47.94°E, 175 m, rainforest, 6.vi.2005 (*B.L.Fisher et al.*); Fianarantsoa, 9.0 km NE Ivohibe, 22.4267°S, 46.9383°E, 900 m, rainforest, 12.xi.1997 (*B.L.Fisher* (*Sylvain*)); Fianarantsoa, Ampangabe I Non Protected Area, 21.4 km W Itremo, 20.6111°S, 46.6069°E, 1414 m, savannah woodland, 21.iii.2010 (*Andrianjaka Ravelomanana*); Fianarantsoa, Antapia I Non Protected Area, 26.43 km SW Ambositra, 20.7197°S, 47.0868°E, 1495 m, Uapaca woodland, 3.ii.2010 (*Andrianjaka Ravelomanana*); Fianarantsoa, Antohatsahomby V Non Protected Area, 22.63 km NW Itremo, 20.5672°S, 46.5792°E, 1726 m, Uapaca woodland, 18.iii.2010 (*Andrianjaka Ravelomanana*); Fianarantsoa, Forêt d’Atsirakambiaty, 7.6 km 285° WNW Itremo, 20.5933°S, 46.5633°E, 1550 m, montane rainforest, 22.i.2003 (*Fisher, Griswold et al.*); Fianarantsoa, Mampiarika I Non Protected Area, 28.08 km SW Ambositra, 20.7344°S, 47.0836°E, 1480 m, Uapaca woodland, 31.i.2010 (*Andrianjaka Ravelomanana*); Fianarantsoa, Mampiarika III Non Protected Area, 28.93 km SW Ambositra, 20.7358°S, 47.084°E, 1487 m, Uapaca woodland, 1.ii.2010 (*Andrianjaka Ravelomanana*); Fianarantsoa, Parc National Befotaka-Midongy, Papango 27.7 km S Midongy-Sud, Mount Papango, 23.8352°S, 46.9637°E, 940 m, rainforest, 13.xi.2006 (*B.L.Fisher et al.*); Fianarantsoa, Parc National d’Isalo, 9.1 km 354° N Ranohira, 22.4817°S, 45.4617°E, 725 m, gallery forest, 27.i.2003 (*Fisher, Griswold et al.*); Fianarantsoa, Parc National de Ranomafana, Vatoharanana River, 4.1 km 231° SW Ranomafana, 21.29°S, 47.4333°E, 1100 m, montane rainforest, 27.iii.2003 (*Fisher, Griswold et al.*); Fianarantsoa, Parc Nationale Ranomafana: Talatakely, 21.2483°S, 47.4267°E, in guava forest, 9.iv.1998 (*CE Griswold, DH Kavanaugh, ND Penny, MJ Raherilalao, JS Ranorianarisoa, J Schwei*); Fianarantsoa, R.S. Ivohibe, 7.5 km ENE Ivohibe, 22.47°S, 46.96°E, 900 m, rainforest, 7.x.1997 (*B.L.Fisher* (*Sylvain*)); Fianarantsoa, Réserve Forestière d’Agnalazaha, Mahabo, 42.9 km 215° Farafangana, 23.1938°S, 47.723°E, 20 m, littoral rainforest, 19.iv.2006 (*B.L. Fisher et al.*); Fianarantsoa, Soanierenana I Non Protected Area, 25.33 km SW Ambositra, 20.7214°S, 47.1099°E, 1723 m, savannah grassland, 6.ii.2010 (*Andrianjaka Ravelomanana*); Mahajanga, Réserve Spéciale Marotandrano, Marotandrano 48.3 km S Mandritsara, 16.2832°S, 48.8144°E, 865 m, transition humid forest, 6.xii.2007 (*B.L.Fisher et al.*); Toamasina, 16 km S Moramanga, 19.0833°S, 48.2333°E, 950 m, rainforest, 18.xi.1990 (*P. S. Ward*); Toamasina, 19 km ESE Maroantsetra, 15.4833°S, 49.9°E, 350 m, rainforest, 22.iv.1989 (*P. S. Ward*); Toamasina, 5.3 km SSE Ambanizana, Andranobe, 15.6713°S, 49.974°E, 425 m, rainforest, 19.xi.1993 (*B.L.Fisher*); Toamasina, 6.3 km S Ambanizana, Andranobe, 15.6813°S, 49.958°E, 25 m, rainforest, 14.xi.1993 (*B.L.Fisher*); Toamasina, 6.9 km NE Ambanizana, Ambohitsitondroina, 15.5851°S, 50.0095°E, 825 m, rainforest, 2.xii.1993 (*B.L.Fisher*); Toamasina, Ambanizana, Parc National Masoala, 15.5717°S, 50.0061°E, 925 m, montane rainforest, 26.ii.2003 (*D. Andriamalala, D. Silva, et al.*); Toamasina, Ambatovy, 12.4 km NE Moramanga, 18.8496°S, 48.2947°E, 1010 m, montane rainforest, 3.iii.2007 (*B.L.Fisher et al.*); Toamasina, Ambatovy, 12.4 km NE Moramanga, 18.8394°S, 48.3084°E, 1080 m, montane rainforest, 4.iii.2007 (*B.L.Fisher et al.*); Toamasina, Analamay, 18.8062°S, 48.3371°E, 1068 m, montane rainforest, 21.iii.2004 (*Malagasy ant team*); Toamasina, Corridor Forestier Analamay-Mantadia, Ambatoharanana, 18.8042°S, 48.4008°E, 968 m, rainforest, 12.xii.2012 (*B.L.Fisher et al.*); Toamasina, Corridor Forestier Analamay-Mantadia, Ambohibolakely, 18.779°S, 48.3638°E, 918 m, rainforest, 23.xi.2012 (*B.L.Fisher et al.*); Toamasina, Corridor Forestier Analamay-Mantadia, Tsaravoniana, 18.7612°S, 48.4213°E, 939 m, rainforest, 2.xii.2012 (*B.L.Fisher et al.*); Toamasina, Corridor Forestier Analamay-Mantadia, Tsaravoniana, 18.7646°S, 48.4194°E, 1039 m, rainforest, 2.xii.2012 (*B.L.Fisher et al.*); Toamasina, Forêt Ambatovy, 14.3 km 57° Moramanga, 18.8508°S, 48.32°E, 1075 m, montane rainforest, 21.iii.2004 (*Malagasy ant team*); Toamasina, Ile Sainte Marie, Forêt Kalalao, 9.9 km 34° Ambodifotatra, 16.9225°S, 49.8873°E, 100 m, rainforest, 24.xi.2005 (*B.L.Fisher et al.*); Toamasina, Montagne d’Akirindro 7.6 km 341° NNW Ambinanitelo, 15.2883°S, 49.5483°E, 600 m, rainforest, 17.iii.2003 (*Fisher, Griswold et al.*); Toamasina, Montagne d’Anjanaharibe, 18.0 km 21° NNE Ambinanitelo, 15.1883°S, 49.615°E, 470 m, rainforest, 8.iii.2003 (*Fisher, Griswold et al.*); Toamasina, Montagne d’Anjanaharibe, 19.5 km 27° NNE Ambinanitelo, 15.1783°S, 49.635°E, 1100 m, montane rainforest, 12.iii.2003 (*Fisher, Griswold et al.*); Toamasina, Parc National de Zahamena, Onibe River, 17.7591°S, 48.8547°E, 780 m, rainforest, 21.ii.2009 (*B.L.Fisher et al.*); Toamasina, Parc National de Zahamena, Tetezambatana forest, near junction of Nosivola and Manakambahiny Rivers, 17.743°S, 48.7294°E, 860 m, rainforest, 18.ii.2009 (*B.L.Fisher et al.*); Toamasina, Parc National Mananara-Nord, 7.1 km 261° Antanambe, 16.455°S, 49.7875°E, 225 m, rainforest, 14.xi.2005 (*B.L.Fisher et al.*); Toamasina, Parcelle K7 Tampolo, 17.2833°S, 49.4167°E, 10 m, littoral forest, 16.iv.2004 (*Malagasy ant team*); Toamasina, Parcelle K9 Tampolo, 17.175°S, 49.268°E, 10 m, littoral forest, 19.iv.2004 (*Malagasy ant team*); Toamasina, Res. Ambodiriana, 4.8 km 306°Manompana, along Manompana river, 16.6723°S, 49.7012°E, 125 m, rainforest, 18.xi.2005 (*B.L.Fisher et al.*); Toamasina, Reserve Betampona, Camp Vohitsivalana, 37.1 km 338° Toamasina, 17.8867°S, 49.2025°E, 520 m, rainforest, 1.xii.2005 (*B.L.Fisher et al.*); Toamasina, Réserve Spéciale Ambatovaky, Sandrangato river, 16.8175°S, 49.295°E, 360 m, rainforest, 25.ii.2010 (*B.L.Fisher et al.*); Toamasina, S.F. Tampolo, 10 km NNE Fenoarivo Atn., 17.2825°S, 49.43°E, 10 m, littoral rainforest, 4.iv.1997 (*B.L.Fisher*); Toamasina, Sahafina forest 11.4 km W Brickaville, 18.8145°S, 48.962°E, 140 m, rainforest, 13.xii.2007 (*B.L.Fisher et al.*); Toamasina, Torotorofotsy, 18.8708°S, 48.3474°E, 1070 m, montane rainforest, marsh edge, 24.iii.2004 (*Malagasy ant team*); Toliara, 10 km NW Enakara, Rés. Andohahela, 24.5667°S, 46.8167°E, 430 m, rainforest, 22.xi.1992 (*B.L.Fisher*); Toliara, 11 km NW Enakara, Rés. Andohahela, 24.5667°S, 46.8333°E, 800 m, rainforest, 17.xi.1992 (*B.L.Fisher*); Toliara, 2.7 km WNW 302° Ste. Luce, 24.7717°S, 47.1717°E, 20 m, littoral rainforest, 9.xii.1998 (*B.L.Fisher* (*J.-Baptiste*)); Toliara, Anosy Region, Distric of Amboasary, 58Km SW of Fort Dauphin, 08 Km NW of Amboasary, Berenty Special Reserve, 25.0067°S, 46.3033°E, 85 m, Galery forest, 25.v.2003 (*Rin’ha, Mike*); Toliara, Forêt Classée d’Analavelona, 29.2 km 343° NNW Mahaboboka, 22.675°S, 44.19°E, 1100 m, montane rainforest, 18.ii.2003 (*Fisher, Griswold et al.*); Toliara, Forêt Classée d’Analavelona, 29.4 km 343° NNW Mahaboboka, 22.675°S, 44.1867°E, 1050 m, montane rainforest, 21.ii.2003 (*Fisher, Griswold et al.*); Toliara, Forêt Ivohibe 55.6 km N Tolagnaro, 24.5617°S, 47.2002°E, 650 m, rainforest, 4.xii.2006 (*B.L.Fisher et al.*); Toliara, Grand Lavasoa, 25.9 km W Tolagnaro, 25.0877°S, 46.749°E, 450 m, rainforest, 30.xi.2006 (*B.L.Fisher et al.*); Toliara, Mandena, 8.4 km NNE 30° Tolagnaro, 24.9517°S, 47.0017°E, 20 m, littoral rainforest, 20.xi.1998 (*B.L.Fisher*); Toliara, Parc National Andohahela, Col de Tanatana, 33.3 km NW Tolagnaro, 24.7585°S, 46.8537°E, 275 m, rainforest, 22.xi.2006 (*B.L.Fisher et al.*); Toliara, Rés. Andohahela, 6 km SSW Eminiminy, 24.7333°S, 46.8°E, 330 m, rainforest, 4.ii.1993 (*P. S. Ward*); Toliara, Réserve Spéciale d’Ambohijanahary, Forêt d’Ankazotsihitafototra, 34.6 km 314° NW Ambaravaranala, 18.26°S, 45.4183°E, 1100 m, montane rainforest, 16.i.2003 (*Fisher, Griswold et al.*); Toliara, Réserve Spéciale d’Ambohijanahary, Forêt d’Ankazotsihitafototra, 35.2 km 312° NW Ambaravaranala, 18.2667°S, 45.4067°E, 1050 m, montane rainforest, 13.i.2003 (*Fisher, Griswold et al.*); Toliara, Réserve Spéciale Kalambatritra, Ampanihy, 23.4635°S, 46.4631°E, 1270 m, montane rainforest, 9.ii.2009 (*B.L.Fisher et al.*); Bongolava Prefec. de Tsiroanomandidy, 6.xii.1974 (*A.Peyrieras*).

### Prionopelta laurae

Taxon classificationPlantaeHymenopteraFormicidae

Overson & Fisher sp. n.

http://zoobank.org/7FC54F7E-4F21-4714-A6C1-D4F6757A7999

Fig. 7

#### Type material.

**Holotype**, pinned worker, MADAGASCAR, Antsiranana, Parc National de Marojejy, Manantenina River, 28.0 km 38° NE Andapa, 8.2 km 333° NNW Manantenina, 14.43667°S, 49.775°E, 450 m, rainforest, sifted litter (leaf mold, rotten wood), collection code: BLF08722, 12.xi.2003 (*B.L. Fisher et al.*) (CASC: CASENT0046149). **Paratypes**, nine pinned workers with same data as holotype (BMNH: CASENT0046153; CASC: CASENT0046141; CASENT0046142; CASENT0046143; CASENT0046147; CASENT0046151; MCZC: CASENT0046150; MHNG: CASENT0046140; NHMB: CASENT0046148).

#### Diagnosis.

*Prionopelta laurae* is the only Malagasy *Prionopelta* with workers that possess nine antennal segments (all others possess twelve). Additionally, it is the smallest of all the Malagasy species with HL < 0.4 mm and HW < 0.3 mm (HL and HW of all other species is greater than 0.4 mm and 0.3 mm respectively).

#### Worker measurements

**(N=16).** HL 0.33–0.38 (0.35); HW 0.24–0.27 (0.26); SL 0.16–0.18 (0.17); WL 0.34–0.4 (0.38); PetL 0.09–0.11 (0.1); PetW 0.13–0.15 (0.14); T1W 0.19–0.23 (0.21); CI 67.55–75.64 (72.71); PI 120.18–152.75 (135.44); SI 61.62–69.05 (65.06).

#### Worker description.

Head much longer than wide with lowest cephalic index on average of all Malagasy *Prionopelta* (mean CI 72.71); posterior head margin straight in full-face view; cephalic foveae small and very dense, with no space for additional cephalic foveae present; median cephalic band devoid of foveae is long and thin and appears slightly swollen or raised above the surrounding integument, forming a scarlike suture; apical tooth of the mandible over four times the length of third tooth in full-face view; nine antennal segments; eye greatly reduced, appearing as a tiny, dark gray patch; majority of marks on pronotum are densely spaced, tiny punctures; mesonotum and propodeum consisting of tiny shallow foveae; metanotal groove visible dorsally; smallest of the *Prionopelta* from Malagasy region; body distinctly pale yellow in color.

#### Etymology.

The name of this species is a patronym dedicated to Laura D. Steger for her continual support during the course of this work and her either completely genuine—or expertly feigned—enthusiasm for being endlessly bombarded by information about ants.

#### Distribution and ecology.

This species has been collected in leaf litter primarily in rainforest with some collections from littoral rainforest and one in tropical dry rainforest, at elevations between 10–600 meters. Its range is restricted to eastern Madagascar and is seemingly disjunct, with most individuals collected from the northeast and only two locales known from the southeast near the coast. No individuals have been collected between Sahafina Forest in the north and Mahabo forest in the south, a distance of 500 km (Fig. 13). The current range of *Prionopelta laurae* is such that it may once have been distributed along the entire eastern coast of Madagascar.

#### Taxonomic notes.

This species of *Prionopelta* is unmistakable as it is the only Malagasy species with nine antennal segments. It is also the smallest known species of Malagasy *Prionopelta* and is a distinct pale yellow color, which is much lighter than the fully-sclerotized workers of any other Malagasy species.

**Figure 7.**
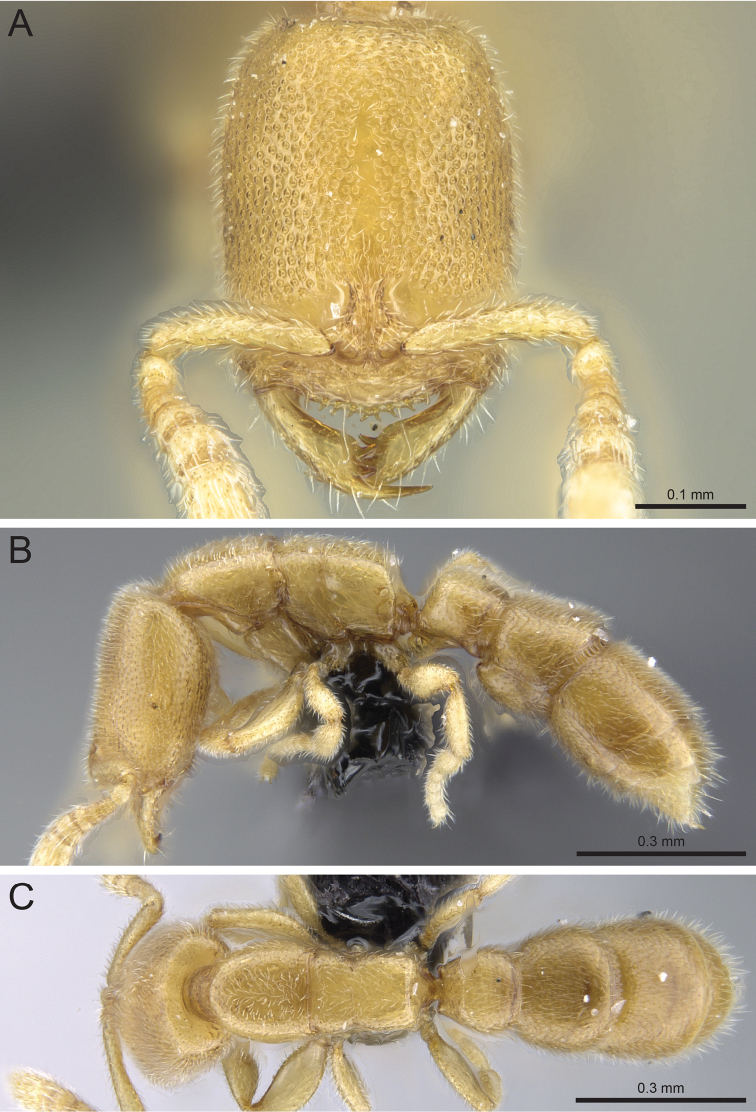
*Prionopelta laurae* paratype worker (CASENT0046151). **A** Head in full-face view **B** Body in profile **C** Body in dorsal view.

#### Non-type material.

MADAGASCAR: Antsiranana, Ambondrobe, 41.1 km 175° Vohemar, 13.7153°S, 50.1017°E, 10 m, littoral rainforest, 29.xi.2004 (*B.L.Fisher*); Antsiranana, Forêt Ambanitaza, 26.1 km 347° Antalaha, 14.6793°S, 50.1837°E, 240 m, rainforest, 26.xi.2004 (*B.L.Fisher*); Antsiranana, Forêt de Binara, 7.5 km 230° SW Daraina, 13.255°S, 49.6167°E, 375 m, tropical dry forest, 1.xii.2003 (*B.L.Fisher*); Fianarantsoa, Réserve Forestière d’Agnalazaha, Mahabo, 42.9 km 215° Farafangana, 23.1938°S, 47.723°E, 20 m, littoral rainforest, 19.iv.2006 (*B.L. Fisher et al.*); Toamasina, 19 km ESE Maroantsetra, 15.4833°S, 49.9°E, 350 m, rainforest, 22.iv.1989 (*P. S. Ward*); Toamasina, Montagne d’Akirindro 7.6 km 341° NNW Ambinanitelo, 15.2883°S, 49.5483°E, 600 m, rainforest, 17.iii.2003 (*Fisher, Griswold et al.*); Toamasina, Nosy Mangabe, 15.5°S, 49.7667°E, 300 m, rainforest, 18.iv.1989 (*P. S. Ward*); Toamasina, Parc National Mananara-Nord, 7.1 km 261° Antanambe, 16.455°S, 49.7875°E, 225 m, rainforest, 14.xi.2005 (*B.L.Fisher et al.*); Toamasina, Reserve Betampona, Camp Rendrirendry 34.1 km 332° Toamasina, 17.924°S, 49.1997°E, 390 m, rainforest, 28.xi.2005 (*B.L.Fisher et al.*); Toamasina, Reserve Betampona, Camp Vohitsivalana, 37.1 km 338° Toamasina, 17.8867°S, 49.2025°E, 520 m, rainforest, 1.xii.2005 (*B.L.Fisher et al.*); Toamasina, Réserve Spéciale Ambatovaky, Sandrangato river, 16.7727°S, 49.2655°E, 450 m, rainforest, 20.ii.2010 (*B.L.Fisher et al.*); Toamasina, Réserve Spéciale Ambatovaky, Sandrangato river, 16.8175°S, 49.295°E, 360 m, rainforest, 25.ii.2010 (*B.L.Fisher et al.*); Toamasina, Sahafina forest 11.4 km W Brickaville, 18.8145°S, 48.962°E, 140 m, rainforest, 13.xii.2007 (*B.L.Fisher et al.*); Toliara, 2.7 km WNW 302° Ste. Luce, 24.7717°S, 47.1717°E, 20 m, littoral rainforest, 9.xii.1998 (*B.L.Fisher, J.-Baptiste*).

### Prionopelta seychelles

Taxon classificationPlantaeHymenopteraFormicidae

Overson & Fisher sp. n.

http://zoobank.org/8DB94B93-AD69-4D75-B5A4-2561EC02622B

Figs 3C , 8

#### Type material.

**Holotype**, pinned worker, SEYCHELLES, Silhouette Island, ridge from Mont Corgat to Mont Cocos Marrons, 4.50126°S, 55.23985°E, 455 m, forest, sifted litter (leaf mold, rotten wood), collection code BLF23364, 24.ii.2010 (*B.L. Fisher et al.*) (CASC: CASENT0161311). **Paratypes**, eight pinned workers with same data as holotype (BMNH: CASENT0161314; CASC: CASENT0161310; CASENT0161312; CASENT0161313; CASENT0161315; CASENT0161316; CASENT0161317; CASENT0161319).

#### Diagnosis.

*Prionopelta seychelles* is the only known species from Seychelles. It can be distinguished from all other Malagasy *Prionopelta* through the following characters: twelve antennal segments; densely arranged cephalic foveae with virtually no space for additional foveae and no shining integument visible between; pronotum consisting of shallow foveae much larger in diameter than those on the head with punctures between; median cephalic band which is devoid of foveae not swelling above the surrounding integument, and often characterized as being wider anteriorly and narrower posteriorly.

#### Worker measurements

**(N=15).** HL 0.44–0.48 (0.46); HW 0.33–0.37 (0.35); SL 0.22–0.26 (0.24); WL 0.47–0.54 (0.5); PetL 0.13–0.17 (0.14); PetW 0.17–0.21 (0.18); T1W 0.29–0.31 (0.3); CI 73.63–80.41 (76.39); PI 106.92–144.44 (128.7); SI 64.56–73.43 (69.77).

#### Worker description.

Twelve antennal segments; posterior margin of the head weakly concave in full-face view; small cephalic foveae densely positioned so that no flat, shining integument is present between; median cephalic band which is devoid of foveae does not appear to swell above the surrounding integument, but rather appears as a smooth, shining surface which is widest anteriorly, narrowing posteriorly; apical tooth intermediate in length; pronotum with foveae which range from shallow to deep and are interspersed regularly with punctures; mesonotum and propodeum consisting of large, shallow foveae; metanotal groove strongly visible, and mesopropodeal suture visible to barely visible, but some depression always present; posterior propodeal edge viewed dorsally is straight or only very slightly concave; no protruding lamellae of the posterior propodeum.

#### Etymology.

This species is named after the Seychelles archipelago, to which it is endemic. The species epithet is a noun in apposition, and thus invariable.

#### Distribution and ecology.

This species is known only from Seychelles and is found between 15–660 meters of elevation on the islands of Mahé, Conception, Thérèse, Silhouette, Praslin, La Digue, Félicité Island, and the Little Sister island group. It does not appear to have strict habitat preferences as it has been collected in mixed forest, littoral forest, non-native forest, palm forest, and coastal scrub. *Prionopelta seychelles* has also been collected from a diversity of microhabitats including from leaf litter, inside rotten logs, under rocks, under moss on live trees, and under root mats.

#### Taxonomic notes.

**Figure 8.**
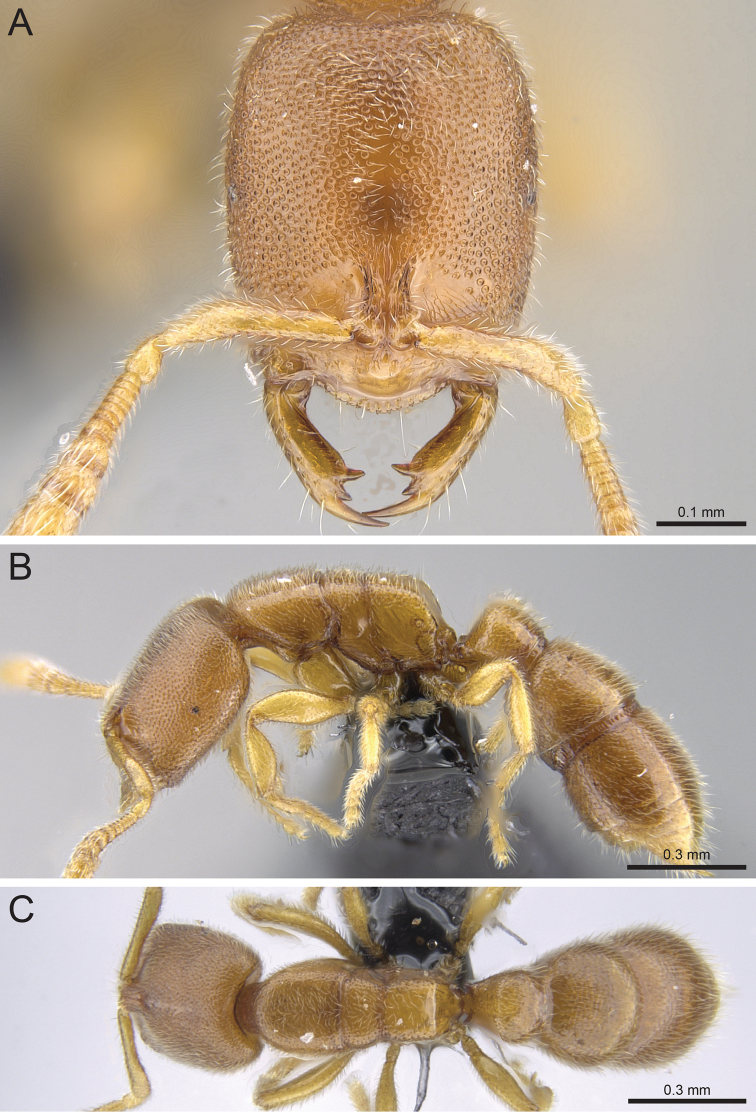
*Prionopelta seychelles* paratype worker (CASENT0161319). **A** Head in full-face view **B** Body in profile **C** Body in dorsal view.

*Prionopelta seychelles* is most likely to be confused with *Prionopelta subtilis*, as both have very small cephalic foveae that are densely arranged on the head. However, the two species are not sympatric, as *Prionopelta seychelles* is known only from Seychelles, and *Prionopelta subtilis* only from Madagascar. The median cephalic bands contrast markedly between these taxa, as that of *Prionopelta seychelles* is wide anteriorly, often tapering posteriorly, with an interrupted border caused by aberrantly placed foveae which break up the margins which define the smooth shining area (Fig. 8A). That of *Prionopelta subtilis*, on the other hand, is very thin throughout its length, with clearly defined borders which are swollen above the surrounding integument (Fig. 3A, B) On average, *Prionopelta subtilis* also has a wider head than *Prionopelta seychelles*: 0.39–0.45 (0.42) vs. 0.33–0.37 (0.35), respectively. Additionally, the cephalic foveae of *Prionopelta subtilis* are smaller and the cephalic sculpture overall appears more delicate than that of *Prionopelta seychelles*.

#### Non-type material.

SEYCHELLES: Conception Island, 4.6631°S, 55.3682°E, 65 m, mixed forest, 12.ii.2010 (*B.L.Fisher et al.*); Félicité Island, 4.3258°S, 55.8698°E, 120 m, forest, 1.ii.2010 (*B.L.Fisher et al.*); La Digue Island, 4.3561°S, 55.8433°E, 300 m, forest, 31.i.2010 (*B.L.Fisher et al.*); La Digue Island, Veuve Réserve, 4.357°S, 55.8279°E, 15 m, littoral forest, 2.ii.2010 (*B.L.Fisher et al.*); Mahé Island, Casse Dent, Morne Seychellois National Park, 4.6528°S, 55.4374°E, 465 m, mixed forest, 11.ii.2010 (*B.L.Fisher et al.*); Mahé Island, Morne Blanc, 4.6574°S, 55.4333°E, 660 m, mixed forest near glacis, 10.ii.2010 (*B.L.Fisher et al.*); Mare Aux Cochon, Mahé Island, 30.vii.2002 (*J. Gerlach*); Praslin Island, 4.3546°S, 55.748°E, 25 m, coastal scrub, 6.ii.2010 (*B.L.Fisher et al.*); Praslin Island, Praslin tower, 4.3409°S, 55.7451°E, 370 m, mixed forest, 3.ii.2010 (*B.L.Fisher et al.*); Praslin Island, Vallée de Mai, 4.331°S, 55.7389°E, 200 m, palm forest, 2.ii.2010 (*B.L.Fisher et al.*); Silhouette Island, above Jardin Marron on crest to Mont Plaisir and Pot à Eau, 4.4867°S, 55.2341°E, 520 m, forest, 20.i.2010 (*B.L.Fisher et al.*); Silhouette Island, Gratte Fesse, 4.4917°S, 55.2389°E, 450 m, forest, 6.iv.2009 (*J. Gerlach*); Silhouette Island, Jardin Marron, 4.4864°S, 55.2363°E, 395 m, non native forest, 27.i.2010 (*B.L.Fisher et al.*); Silhouette Island, Jardin Marron, 4.4864°S, 55.2364°E, 390 m, 24.iii.2009 (*J. Gerlach*); Silhouette Island, La Passe, 4.4847°S, 55.2508°E, 20 m, park/garden, 12.vii.2001 (*J. Gerlach*); Silhouette Island, on path to Anse Mondon, 4.4689°S, 55.2294°E, 255 m, forest, 23.i.2010 (*B.L.Fisher et al.*); Silhouette Island, on plateau toward Gratte Fesse, 4.4879°S, 55.2342°E, 490 m, forest, 22.i.2010 (*B.L.Fisher et al.*).

### Prionopelta subtilis

Taxon classificationPlantaeHymenopteraFormicidae

Overson & Fisher sp. n.

http://zoobank.org/A5A3CDAC-71AF-48E1-914A-5086872539F0

Figs 3A, B , 9

#### Type material.

**Holotype**, pinned worker, MADAGASCAR, Toamasina, Montagne d’Anjanaharibe, 18.0 km 21° NNE Ambinanitelo, 15.18833°S, 49.615°E, 470 m, rainforest, sifted litter (leaf mold, rotten wood), BLF08002, 8.iii.2003 (*B.L. Fisher et al.*) (CASC: CASENT0033641). **Paratypes**, 23 pinned workers with same data as holotype (BMNH: CASENT0033585; CASC: CASENT0033582; CASENT0033586; CASENT0033588; CASENT0033590; CASENT0033591; CASENT0033596; CASENT0033597; CASENT0033598; CASENT0033599; CASENT0033600; CASENT0033601; CASENT0033603; CASENT0033606; CASENT0033610; CASENT0033611; CASENT0033613; CASENT0033614; CASENT0033615; CASENT0033644; MCZC: CASENT0033604; MHNG: CASENT0033584; NHMB: CASENT0033643).

#### Diagnosis.

*Prionopelta subtilis* can be recognized by the following combination of characters: twelve antennal segments; minute, densely placed cephalic foveae with raised margins where foveae touch so that the entire head is covered in a delicate mosaic of connected foveae with ridges between; well-defined, uniformly narrow, coronal suture that swells above the level of the surrounding integument; shallow foveae on the pronotum are much larger than those on the head, and more widely spaced, with tiny punctures between.

#### Worker measurements

**(N=20).** HL 0.47–0.57 (0.52); HW 0.39–0.45 (0.42); SL 0.26–0.32 (0.29); WL 0.5–0.67 (0.58); PetL 0.14–0.18 (0.16); PetW 0.19–0.26 (0.22); T1W 0.31–0.4 (0.35); CI 76.55–86.41 (80.5); PI 126.32–150.69 (139.98); SI 61.34–71.39 (68.14).

#### Worker description.

Posterior head margin slightly concave with a noticeable notch medially; cephalic foveae dense and minute; virtually no area of the head lacking foveae in full-face view except at the extreme posterolateral corners; median cephalic band devoid of foveae is thin, linear, and slightly but uniformly swells above the surrounding integument; apical tooth intermediate in length; foveae on the pronotum are shallow, as well as more widely spaced and obviously larger than those on the head, with punctures present between; mesonotum and propodeum consisting of shallow foveae and punctures; metanotal groove visible dorsally, and mesopropodeal suture strongly visible in lateral view.

#### Etymology.

The name of this species comes from the Latin adjective meaning “fine”, “thin”, or “slender” and refers to the very delicate, net-like patterns produced by the sculpture on the head.

#### Distribution and ecology.

This common and widespread species is found in rainforest, montane rainforest, lowland rainforest, tropical forest, littoral forest, degraded forest, and marsh edge from 5–1325 meters of elevation (Fig. 13). On the ground it has been collected from inside rotten logs and sticks, as well as under moss, rocks, logs, and in litter. It has also been collected from above-ground sites including canopy moss and leaf litter, as well as inside above-ground twigs and branches.

#### Taxonomic notes.

*Prionopelta subtilis* is easy to recognize at a glance under high magnification once several individuals have been observed, as the very small and delicate foveae covering the entire surface of the head produce a unique visual appearance among the Malagasy *Prionopelta*. The only other confusable species with dense, directly adjacent foveae across the entire head (besides *Prionopelta laurae*, which would not be mistaken for *Prionopelta subtilis*) are *Prionopelta seychelles*, and some *Prionopelta descarpentriesi*. Unlike *Prionopelta subtilis*, *Prionopelta seychelles* does not possesses a coronal suture medially on the head. Some individuals of *Prionopelta descarpentriesi* have a dense pattern of touching foveae with a network of ridges between them across the entire head, but these foveae are much larger and deeper than in *Prionopelta subtilis*. Additionally, this trait in *Prionopelta descarpentriesi* is accompanied by sculpture on the pronotum that consists almost entirely of large foveae which are similar in size to those on the head, whereas *Prionopelta subtilis* has much larger foveae on its pronotum than on its head, and these foveae are interspersed with punctures.

**Figure 9.**
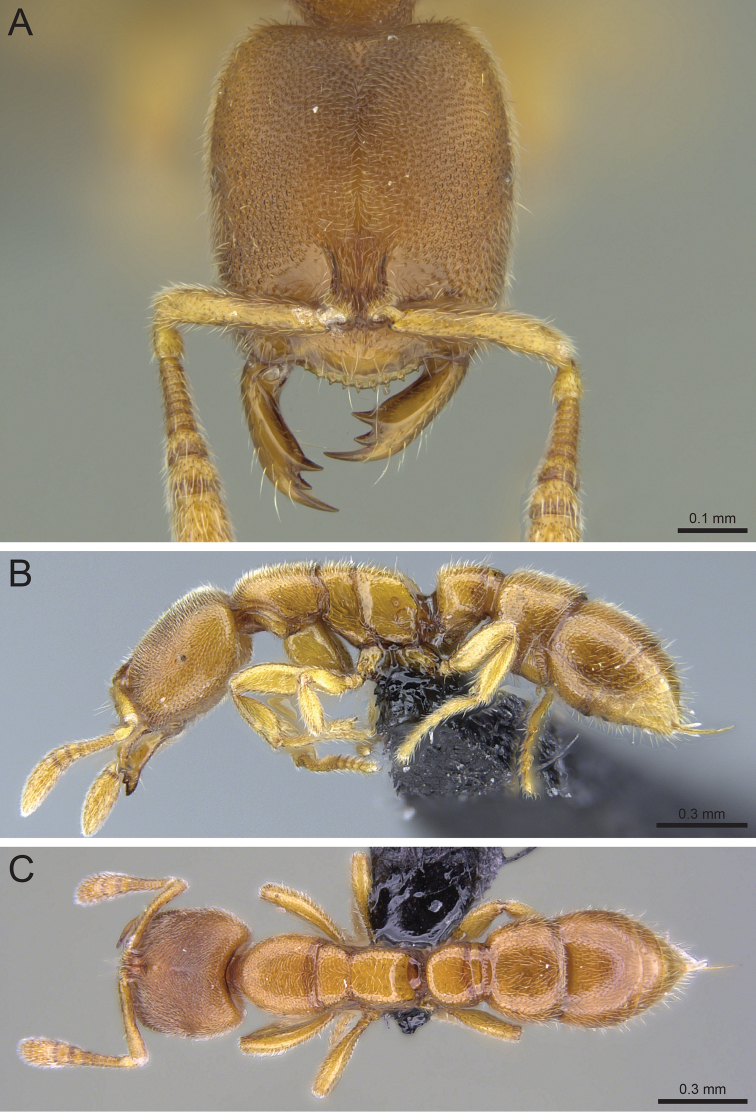
*Prionopelta subtilis* holotype worker (CASENT0033641). **A** Head in full-face view **B** Body in profile **C** Body in dorsal view.

#### Non-type material.

MADAGASCAR: Andasibe, Mantadia NP, 7.i.2006 (*A.Ballerio*); Antsiranana, 6.5 km SSW Befingotra, Rés. Anjanaharibe-Sud, 14.75°S, 49.5°E, 875 m, rainforest, 19.x.1994 (*B.L.Fisher*); Antsiranana, 9.2 km WSW Befingotra, Rés. Anjanaharibe-Sud, 14.75°S, 49.4667°E, 1200 m, montane rainforest, 9.xi.1994 (*B.L.Fisher*); Antsiranana, Betaolana Forest, along Bekona River, 14.53°S, 49.4404°E, 880 m, rainforest, 4.iii.2009 (*B.L.Fisher et al.*); Antsiranana, Binara Forest, 13.2639°S, 49.5992°E, 1065 m, rainforest, 18.x.2013 (*B.L.Fisher et al.*); Antsiranana, Forêt de Binara, 9.4 km 235° SW Daraina, 13.2633°S, 49.6°E, 1100 m, montane rainforest, 5.xii.2003 (*B.L.Fisher*); Antsiranana, Makirovana forest, 14.1651°S, 49.9477°E, 900 m, montane rainforest, 30.iv.2011 (*B.L.Fisher et al.*); Antsiranana, Makirovana forest, 14.1667°S, 49.95°E, 715 m, rainforest, 2.v.2011 (*B.L.Fisher et al.*); Antsiranana, Makirovana forest, 14.1604°S, 49.9522°E, 550 m, rainforest, 1.v.2011 (*B.L.Fisher et al.*); Antsiranana, Makirovana forest, 14.1707°S, 49.9541°E, 415 m, rainforest, 29.iv.2011 (*B.L.Fisher et al.*); Antsiranana, Parc National de Marojejy, Antranohofa, 26.6 km 31° NNE Andapa, 10.7 km 318° NW Manantenina, 14.4433°S, 49.7433°E, 1325 m, montane rainforest, 18.xi.2003 (*B.L.Fisher*); Antsiranana, Parc National de Marojejy, Manantenina River, 27.6 km 35° NE Andapa, 9.6 km 327° NNW Manantenina, 14.435°S, 49.76°E, 775 m, rainforest, 15.xi.2003 (*B.L.Fisher et al.*); Antsiranana, Parc National de Marojejy, Manantenina River, 28.0 km 38° NE Andapa, 8.2 km 333° NNW Manantenina, 14.4367°S, 49.775°E, 450 m, rainforest, 12.xi.2003 (*B.L.Fisher et al.*); Antsiranana, Parc National Montagne d’Ambre, 12.5178°S, 49.1796°E, 1000 m, montane rainforest, 4.iii.2011 (*B.L.Fisher et al.*); Antsiranana, Parc National Montagne d’Ambre, 12.5139°S, 49.1778°E, 984 m, montane rainforest, 25.ii.2011 (*B.L.Fisher et al.*); Antsiranana, Parc National Montagne d’Ambre, 12.5231°S, 49.179°E, 1100 m, montane rainforest, 11.iii.2011 (*B.L.Fisher et al.*); Antsiranana, Parc National Montagne d’Ambre, 12.5342°S, 49.1761°E, 1325 m, montane rainforest, 12.iii.2011 (*B.L.Fisher et al.*); Antsiranana, Parc National Montagne d’Ambre, 12.5247°S, 49.1724°E, 1235 m, montane rainforest, 10.iii.2011 (*B.L.Fisher et al.*); Antsiranana, Parc National Montagne d’Ambre, 3.6 km 235° SW Joffreville, 12.5344°S, 49.1795°E, 925 m, montane rainforest, 20.i.2001 (*Fisher, Griswold et al.*); Antsiranana, Parc National Montagne d’Ambre, Mahasarika, 12.5318°S, 49.1766°E, 1135 m, montane rainforest, 19.xi.2007 (*B.L.Fisher et al.*); Antsiranana, Parc National Montagne d’Ambre, Petit lac, 12.5366°S, 49.1741°E, 1130 m, montane rainforest, 17.xi.2007 (*B.L.Fisher et al.*); Antsiranana, Parc National Montagne d’Ambre, Roussettes, 12.5257°S, 49.1724°E, 1025 m, montane rainforest, 15.xi.2007 (*B.L.Fisher et al.*); Fianarantsoa, 43 km S Ambalavao, Rés. Andringitra, 22.2333°S, 47°E, 825 m, rainforest, 5.x.1993 (*B.L.Fisher*); Fianarantsoa, 45 km S. Ambalavao, 22.2167°S, 47.0167°E, 785 m, rainforest, 25.ix.1993 (*B.L.Fisher*); Fianarantsoa, Forêt d’Ambalagoavy Nord, Ikongo, Ambatombe, 21.8275°S, 47.3389°E, 625 m, 1.xii.2000 (*R. Harin’Hala & M.E. Irwin*); Fianarantsoa, Forêt de Vevembe, 66.6 km 293° Farafangana, 22.791°S, 47.1818°E, 600 m, rainforest, transition to montane forest, 24.iv.2006 (*B.L. Fisher et al.*); Fianarantsoa, Réserve Forestière d’Agnalazaha, Mahabo, 42.9 km 215° Farafangana, 23.1938°S, 47.723°E, 20 m, littoral rainforest, 19.iv.2006 (*B.L. Fisher et al.*); Fianarantsoa, Réserve Spéciale Manombo 24.5 km 228° Farafangana, 23.0158°S, 47.719°E, 30 m, rainforest, 22.iv.2006 (*B.L. Fisher et al.*); Mahajanga, Réserve Spéciale Marotandrano, Marotandrano 48.3 km S Mandritsara, 16.2832°S, 48.8144°E, 865 m, transition humid forest, 7.xii.2007 (*B.L.Fisher et al.*); Toamasina, 16 km S Moramanga, 19.0833°S, 48.2333°E, 950 m, rainforest, 18.xi.1990 (*P. S. Ward*); Toamasina, 5.3 km SSE Ambanizana, Andranobe, 15.6713°S, 49.974°E, 425 m, rainforest, 21.xi.1993 (*B.L.Fisher*); Toamasina, 6.3 km S Ambanizana, Andranobe, 15.6813°S, 49.958°E, 25 m, rainforest, 14.xi.1993 (*B.L.Fisher*); Toamasina, 6.9 km NE Ambanizana, Ambohitsitondroina, 15.5851°S, 50.0095°E, 825 m, rainforest, 2.xii.1993 (*B.L.Fisher*); Toamasina, 6.9 km NE Ambanizana, Ambohitsitondroina, 15.5667°S, 50°E, 1000 m, montane rainforest, 8.xii.1993 (*B.L.Fisher*); Toamasina, 6 km ESE Andasibe (=Perinet), 18.95°S, 48.4667°E, 900 m, rainforest, 17.xi.1990 (*P. S. Ward*); Toamasina, Ambanizana, Parc National Masoala, 15.5717°S, 50.0061°E, 925 m, montane rainforest, 26.ii.2003 (*D. Andriamalala, D. Silva, et al.*); Toamasina, Ambanizana, Parc National Masoala, 15.5722°S, 50.0069°E, 1020 m, montane rainforest, 2.iii.2003 (*D. Andriamalala, D. Silva, et al.*); Toamasina, Ambatovy, 12.4 km NE Moramanga, 18.8394°S, 48.3084°E, 1080 m, montane rainforest, 8.iii.2007 (*B.L.Fisher et al.*); Toamasina, Ambatovy, 12.4 km NE Moramanga, 18.8496°S, 48.2947°E, 1010 m, montane rainforest, 3.iii.2007 (*B.L.Fisher et al.*); Toamasina, Analamay, 18.8062°S, 48.3371°E, 1068 m, montane rainforest, 21.iii.2004 (*Malagasy ant team*); Toamasina, Ankerana, 18.4067°S, 48.8228°E, 681 m, degraded forest, 28.i.2012 (*B.L.Fisher et al.*); Toamasina, Ankerana, 18.4064°S, 48.8025°E, 1108 m, montane forest, 19.i.2012 (*B.L.Fisher et al.*); Toamasina, Ankerana, 18.4104°S, 48.8189°E, 855 m, rainforest, 25.i.2012 (*B.L.Fisher et al.*); Toamasina, Ankerana, 18.4017°S, 48.806°E, 1035 m, montane forest, 24.i.2012 (*B.L.Fisher et al.*); Toamasina, Ankerana, 18.4006°S, 48.8131°E, 865 m, rainforest, 17.i.2012 (*B.L.Fisher et al.*); Toamasina, Ankerana, 18.4083°S, 48.8211°E, 750 m, rainforest, 21.i.2012 (*B.L.Fisher et al.*); Toamasina, Ankerana, 18.4061°S, 48.8203°E, 725 m, rainforest, 16.i.2012 (*B.L.Fisher et al.*); Toamasina, Bevolota 17.1 km N Andasibe, 18.7707°S, 48.4316°E, 995 m, montane rainforest, 12.xii.2007 (*B.L.Fisher et al.*); Toamasina, Corridor Forestier Analamay-Mantadia, Ambatoharanana, 18.8042°S, 48.4008°E, 968 m, rainforest, 12.xii.2012 (*B.L.Fisher et al.*); Toamasina, Corridor Forestier Analamay-Mantadia, Ambatoharanana, 18.8039°S, 48.4051°E, 1013 m, rainforest, 12.xii.2012 (*B.L.Fisher et al.*); Toamasina, Corridor Forestier Analamay-Mantadia, Ambatoharanana, 18.804°S, 48.4036°E, 1064 m, rainforest, 12.xii.2012 (*B.L.Fisher et al.*); Toamasina, Corridor Forestier Analamay-Mantadia, Ambatoharanana, 18.8044°S, 48.4074°E, 960 m, rainforest, 12.xii.2012 (*B.L.Fisher et al.*); Toamasina, Corridor Forestier Analamay-Mantadia, Ambohibolakely, 18.779°S, 48.3638°E, 918 m, rainforest, 23.xi.2012 (*B.L.Fisher et al.*); Toamasina, Corridor Forestier Analamay-Mantadia, Ambohibolakely, 18.7791°S, 48.3663°E, 1014 m, rainforest, 23.xi.2012 (*B.L.Fisher et al.*); Toamasina, Corridor Forestier Analamay-Mantadia, Ambohibolakely, 18.7609°S, 48.3713°E, 1044 m, rainforest, 29.xi.2012 (*B.L.Fisher et al.*); Toamasina, Corridor Forestier Analamay-Mantadia, Ambohibolakely, 18.7613°S, 48.3644°E, 983 m, rainforest, 26.xi.2012 (*B.L.Fisher et al.*); Toamasina, Corridor Forestier Analamay-Mantadia, Tsaravoniana, 18.7612°S, 48.4213°E, 939 m, rainforest, 2.xii.2012 (*B.L.Fisher et al.*); Toamasina, Corridor Forestier Analamay-Mantadia, Tsaravoniana, 18.7637°S, 48.4203°E, 984 m, rainforest, 2.xii.2012 (*B.L.Fisher et al.*); Toamasina, Corridor Forestier Analamay-Mantadia, Tsaravoniana, 18.7646°S, 48.4194°E, 1039 m, rainforest, 4.xii.2012 (*B.L.Fisher et al.*); Toamasina, Corridor Forestier Analamay-Mantadia, Tsaravoniana, 18.7574°S, 48.423°E, 1018 m, rainforest, 8.xii.2012 (*B.L.Fisher et al.*); Toamasina, F.C. Andriantantely, 18.695°S, 48.8133°E, 530 m, rainforest, 4.xii.1998 (*H.J.Ratsirarson*); Toamasina, F.C. Didy, 18.1983°S, 48.5783°E, 960 m, rainforest, 16.xii.1998 (*H.J.Ratsirarson*); Toamasina, F.C. Sandranantitra, 18.0483°S, 49.0917°E, 450 m, rainforest, 18.i.1999 (*H.J.Ratsirarson*); Toamasina, Forêt Ambatovy, 14.3 km 57° Moramanga, 18.8508°S, 48.32°E, 1075 m, montane rainforest, 12.iv.2005 (*B.L.Fisher*); Toamasina, Montagne d’Akirindro 7.6 km 341° NNW Ambinanitelo, 15.2883°S, 49.5483°E, 600 m, rainforest, 17.iii.2003 (*Fisher, Griswold et al.*); Toamasina, Montagne d’Anjanaharibe, 18.0 km 21° NNE Ambinanitelo, 15.1883°S, 49.615°E, 470 m, rainforest, 8.iii.2003 (*Fisher, Griswold et al.*); Toamasina, Montagne d’Anjanaharibe, 19.5 km 27° NNE Ambinanitelo, 15.1783°S, 49.635°E, 1100 m, montane rainforest, 12.iii.2003 (*Fisher, Griswold et al.*); Toamasina, Nosy Mangabe, 7.43 km S Maroantsetra, 15.4973°S, 49.7622°E, 5 m, littoral rainforest edge, 25.vii.2007 (*B.L.Fisher et al.*); Toamasina, P.N. Mantadia, 18.7917°S, 48.4267°E, 895 m, rainforest, 25.xi.1998 (*H.J.Ratsirarson*); Toamasina, Parc National de Zahamena, 17.7336°S, 48.7263°E, 950 m, rainforest, 19.ii.2009 (*B.L.Fisher et al.*); Toamasina, Parc National de Zahamena, Besaky River, 17.7524°S, 48.8532°E, 760 m, rainforest, 22.ii.2009 (*B.L.Fisher et al.*); Toamasina, Parc National de Zahamena, Onibe River, 17.7591°S, 48.8547°E, 780 m, rainforest, 21.ii.2009 (*B.L.Fisher et al.*); Toamasina, Parc National de Zahamena, Sahavorondrano River, 17.7526°S, 48.8573°E, 765 m, rainforest, 23.ii.2009 (*B.L.Fisher et al.*); Toamasina, Parc National de Zahamena, Tetezambatana forest, near junction of Nosivola and Manakambahiny Rivers, 17.743°S, 48.7294°E, 860 m, rainforest, 18.ii.2009 (*B.L.Fisher et al.*); Toamasina, Reserve Betampona, Camp Rendrirendry 34.1 km 332° Toamasina, 17.924°S, 49.1997°E, 390 m, rainforest, 29.xi.2005 (*B.L.Fisher et al.*); Toamasina, Reserve Betampona, Camp Vohitsivalana, 37.1 km 338° Toamasina, 17.8867°S, 49.2025°E, 520 m, rainforest, 2.xii.2005 (*B.L.Fisher et al.*); Toamasina, Réserve Nationale Intégrale Betampona, Betampona 35.1 km NW Toamasina, 17.918°S, 49.2007°E, 500 m, rainforest, 15.xii.2007 (*B.L.Fisher et al.*); Toamasina, Réserve Spéciale Ambatovaky, Sandrangato river, 16.7702°S, 49.2664°E, 470 m, rainforest, 23.ii.2010 (*B.L.Fisher et al.*); Toamasina, Réserve Spéciale Ambatovaky, Sandrangato river, 16.7747°S, 49.2655°E, 355 m, rainforest along river, 21.ii.2010 (*B.L.Fisher et al.*); Toamasina, Réserve Spéciale Ambatovaky, Sandrangato river, 16.7633°S, 49.2669°E, 520 m, rainforest, 22.ii.2010 (*B.L.Fisher et al.*); Toamasina, Réserve Spéciale Ambatovaky, Sandrangato river, 16.7727°S, 49.2655°E, 450 m, rainforest, 20.ii.2010 (*B.L.Fisher et al.*); Toamasina, Réserve Spéciale Ambatovaky, Sandrangato river, 16.7691°S, 49.267°E, 475 m, rainforest, 21.ii.2010 (*B.L.Fisher et al.*); Toamasina, Réserve Spéciale Ambatovaky, Sandrangato river, 16.8175°S, 49.295°E, 360 m, rainforest, 25.ii.2010 (*B.L.Fisher et al.*); Toamasina, S.F. Tampolo, 10 km NNE Fenoarivo Atn., 17.2825°S, 49.43°E, 10 m, littoral rainforest, 5.iv.1997 (*B.L.Fisher*); Toamasina, Sahafina forest 11.4 km W Brickaville, 18.8145°S, 48.962°E, 140 m, rainforest, 13.xii.2007 (*B.L.Fisher et al.*); Toamasina, Torotorofotsy, 18.8708°S, 48.3474°E, 1070 m, montane rainforest, marsh edge, 24.iii.2004 (*Malagasy ant team*); Toamasina, Torotorofotsy, 18.7705°S, 48.4304°E, 1005 m, montane rainforest, 12.iii.2012 (*B.L.Fisher et al.*); Toliara, Parc National Andohahela, Col de Tanatana, 33.3 km NW Tolagnaro, 24.7585°S, 46.8537°E, 275 m, rainforest, 28.xi.2006 (*B.L.Fisher et al.*); Toliara, Parc National d’Andohahela, Manampanihy River, 5.4 km 113° ESE Mahamavo, 36.7 km 343° NNW Tolagnaro, 24.7639°S, 46.7668°E, 650 m, rainforest, 24.i.2002 (*Fisher-Griswold Arthropod Team*); Toliara, Rés. Andohahela, 6 km SSW Eminiminy, 24.7333°S, 46.8°E, 330 m, rainforest, 4.ii.1993 (*P.S.Ward*); Toliara, Réserve Spéciale d’Ambohijanahary, Forêt d’Ankazotsihitafototra, 35.2 km 312° NW Ambaravaranala, 18.2667°S, 45.4067°E, 1050 m, montane rainforest, 13.i.2003 (*Fisher, Griswold et al.*).

### Prionopelta talos

Taxon classificationPlantaeHymenopteraFormicidae

Overson & Fisher sp. n.

http://zoobank.org/D7559E6C-40E3-47A5-B43A-5D5E14828003

Figs 4A, D , 10

#### Type material.

**Holotype**, pinned worker, MADAGASCAR, Antsiranana, 9.2 km WSW Befingotra, Rés. Anjanaharibe-Sud, 14.75°S, 49.46667°E, 1260 m, montane rainforest, canopy moss and leaf litter, collection code BLF01217, 11.xi.1994 (*B.L. Fisher et al.*) (CASC: CASENT0009472). **Paratypes**, six pinned workers with same data as holotype (CASC: CASENT0009473; CASENT0009474; CASENT0191885; CASENT0339228; CASENT0339229; CASENT0339230).

#### Diagnosis.

*Prionopelta talos* can be recognized by the following combination of characters: twelve antennal segments; large globular eye which appears as a half-sphere emerging from the surface of the head, composed of several asymmetrical subunits visible under high magnification; pronounced, tricolored body with a dark brown head, light brown body, and yellow/pale legs; known only from the Anjanaharibe-Sud Reserve in northeastern Madagascar.

#### Worker measurements

**(N=9).** HL 0.5–0.53 (0.52); HW 0.39–0.43 (0.41); SL 0.28–0.3 (0.28); WL 0.52–0.62 (0.56); PetL 0.14–0.16 (0.15); PetW 0.21–0.24 (0.23); T1W 0.35–0.38 (0.36); CI 77.07–81.19 (79.06); PI 143.95–163.83 (152.84); SI 67.23–72.59 (69.8).

#### Worker description.

In full-face view, cephalic foveae become denser medially, so that laterally, foveae are separated by more than the diameter of one fovea, and medially, many foveae are directly adjacent and tend to form longitudinal chains of connected foveae; areas between foveae dark brown and shining; median cephalic area devoid of foveae not swelling above the surface of the surrounding integument and characterized as being widest anteriorly, narrowing posteriorly; apical tooth relatively short in length; eyes largest of all Malagasy *Prionopelta*, uniformly globular in shape, and under very high magnification, appear to be composed of several subunits which form a conglomerate half-sphere; both large, shallow foveae and small punctures present on pronotum and mesonotum; propodeal surface possesses only large shallow foveae; strong metanotal groove in dorsal view; strong mesopropodeal suture; distinctly tricolored body with uniformly dark brown head, tan mesosoma and gaster, and pale yellow legs and antennae.

#### Etymology.

The rich brown color of the shiny integument of this taxon inspired the name “Talos”, after the living bronze statue that protected Europa from invaders in Greek mythology. The species epithet is an arbitrary combination of letters, and thus invariant.

#### Distribution and ecology.

This rare ant is known from a single locality in the Anjanaharibe-Sud Reserve in the province of Antsiranana (Fig. 13). It is found in montane rainforest at an elevation of 1260 meters.

#### Taxonomic notes.

*Prionopelta talos* is most likely to be confused with *Prionopelta descarpentriesi* as it has a similar arrangement of cephalic foveae to some individuals of *Prionopelta descarpentriesi* (morphotype A). However, *Prionopelta talos* has distinctly shaped and larger eyes and a striking color pattern that distinguishes it quite easily from all Malagasy congeners.

**Figure 10.**
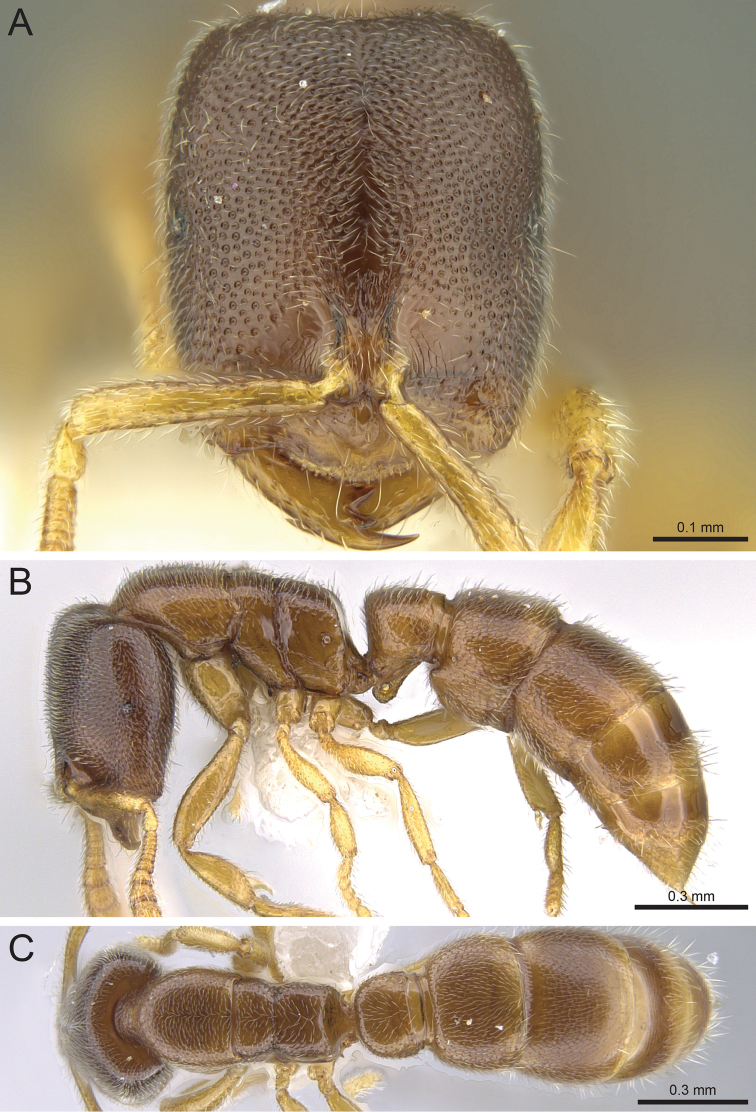
*Prionopelta talos* holotype worker (CASENT0009472). **A** Head in full-face view **B** Body in profile **C** Body in dorsal view.

### Prionopelta vampira

Taxon classificationPlantaeHymenopteraFormicidae

Overson & Fisher sp. n.

http://zoobank.org/4B5C917A-C3A8-4CFC-8E25-0F50321B0F67

**Figure 11.**
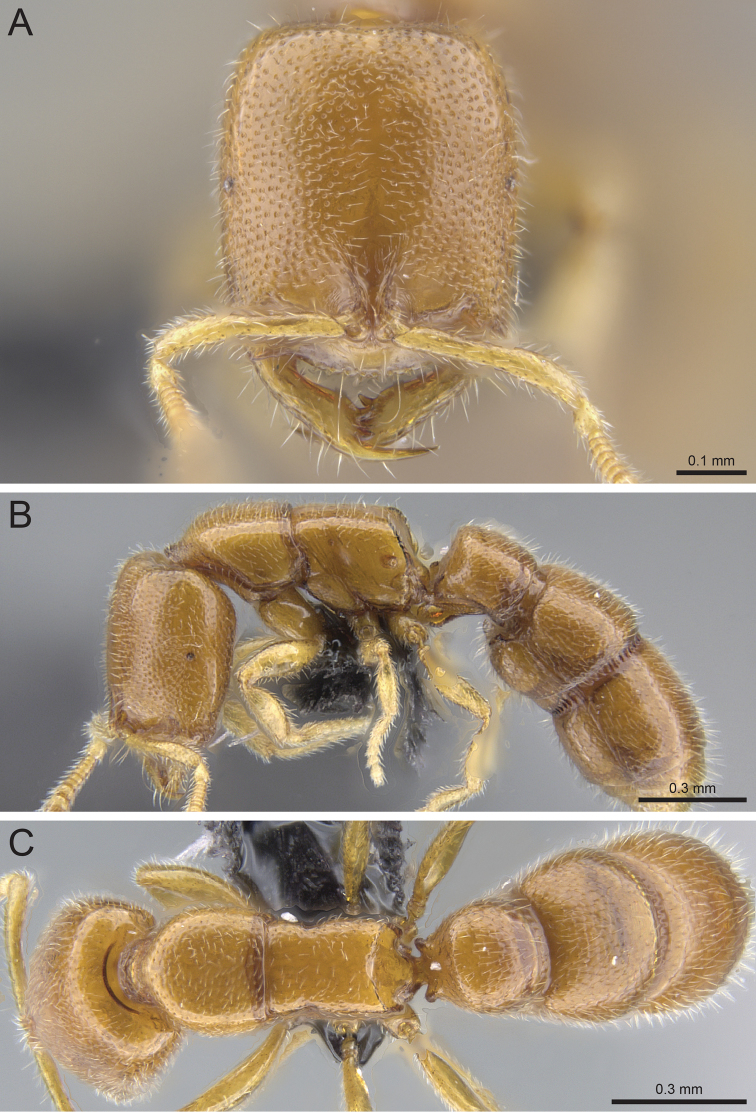
*Prionopelta vampira* holotype worker (CASENT0041504). **A** Head in full-face view **B** Body in profile **C** Body in dorsal view.

Figs 1B , 2A, C , 11

#### Type material.

**Holotype**, pinned worker, MADAGASCAR, Antsiranana, Forêt d’Analabe, 30.0 km 72° ENE Daraina, 13.08333°S, 49.90833°E, 30 m, littoral rainforest, sifted litter (leaf mold, rotten wood), collection code BLF09426, 27.xi.2003 (*B.L. Fisher et al.*) (CASC: CASENT0041504). **Paratypes**, three pinned workers with same data as holotype (CASC: CASENT0041500; CASENT0041501; CASENT0041502).

#### Diagnosis.

*Prionopelta vampira* is the only member of the genus from the Malagasy region in which workers entirely lack any visible metanotal suture when viewed dorsally; additionally the posterior propodeal edge is noticeably more concave in dorsal view than any other Malagasy *Prionopelta*.

#### Worker measurements

**(N=8).** HL 0.49–0.53 (0.5); HW 0.41–0.45 (0.42); SL 0.27–0.31 (0.28); WL 0.55–0.62 (0.58); PetL 0.19–0.23 (0.21); PetW 0.24–0.26 (0.25); T1W 0.35–0.39 (0.37); CI 81.78–85.31 (83.78); PI 103.96–130.2 (120.71); SI 64.25–69.36 (67.33).

#### Worker description.

Highest cephalic index on average of Malagasy *Prionopelta* (CI 81.78–85.31 (83.78); posterior margin of the head with slight notch medially in full-face view; cephalic foveae shallow, large, and widely spaced; directly adjacent cephalic foveae either completely lacking, or very rare; if any foveae are adjacent, then always with only 2–3 foveae connected, and these usually always medially on the head in full-face view; majority of cephalic foveae separated by 1–3 foveal diameters, appear cleanly scooped from the shining integument, and lack raised margins; median cephalic band devoid of foveae is uniformly broad, and not swelling above the integument; apical tooth very long, longest of all Malagasy *Prionopelta*, over four times the length of the third apical tooth measured from base to tip (Fig. 2C); sculpture of the dorsum of the mesosoma consisting of large, shallow foveae which are widely spaced at 2–3 foveal diameters with punctures present between foveae; no metanotal suture present in dorsal view, but rather a shining surface with no clear distinction between propodeum and mesonotum; in a few specimens, a slightly perceptible depression is sometimes visible at the site of the metanotal suture, with associated notches on the lateral edges of the dorsum of the mesosoma, but this depression always lacks scarring; in lateral view, mesopropodeal suture weak, appearing as a gradual depression rather than a scar; posterior propodeal edge seen dorsally strongly concave; sharp lamellae of the posterior propodeum present.

#### Etymology.

The name of this species is inspired by the vampire-like nature of its exceptionally long apical tooth. The species epithet is a Latinized adjective of the German and Hungarian word “vampir”.

#### Distribution and ecology.

This species is almost entirely restricted to northern Madagascar where it is found in litter in rainforest, littoral rainforest, and montane rainforest from 25–1200 meters of elevation. Intriguingly, *Prionopelta vampira* has also been collected at a single, highly disjunct site in far southeastern Madagascar 1048 km to the south near Enakara in the province of Toliara (Fig. 13).

#### Taxonomic notes.

This species, which has similar cephalic scupturing to *Prionopelta xerosilva*, is otherwise unmistakable due to its extremely long apical tooth, lack of a metanotal suture, and strongly concave posterior propodeal edge when viewed dorsally.

#### Non-type material.

MADAGASCAR: Antsiranana, Ampasindava, Andranomatavy Forest, 13.669°S, 47.9877°E, 149 m, disturbed dry forest, 6.x.2013 (*B.L.Fisher et al.*); Antsiranana, Ampasindava, Andranomatavy Forest, 13.663°S, 47.9794°E, 543 m, disturbed dry forest, 6.x.2013 (*B.L.Fisher et al.*); Antsiranana, Forêt Ambato, 26.6 km 33° Ambanja, 13.4645°S, 48.5517°E, 150 m, rainforest, 8.xii.2004 (*B.L.Fisher*); Antsiranana, Forêt d’ Andavakoera, 21.4 km 75° ENE Ambilobe; 4.6 km 356° N Betsiaka, 13.1183°S, 49.23°E, 425 m, rainforest, 15.xii.2003 (*B.L.Fisher*); Antsiranana, Forêt d’Ampombofofo, 12.0995°S, 49.3387°E, 25 m, littoral forest, 21.xi.2007 (*B.L.Fisher et al.*); Antsiranana, Forêt d’Analabe, 30.0 km 72° ENE Daraina, 13.0833°S, 49.9083°E, 30 m, littoral rainforest, 27.xi.2003 (*B.L.Fisher*); Antsiranana, Makirovana forest, 14.1707°S, 49.9541°E, 225 m, rainforest, 4.v.2011 (*B.L.Fisher et al.*); Antsiranana, Nosy Be, Réserve Naturelle Intégrale de Lokobe, 6.3 km 112° ESE Hellville, 13.4193°S, 48.3312°E, 30 m, rainforest, 19.iii.2001 (*Fisher, Griswold et al.*); Toliara, 11 km NW Enakara, Rés. Andohahela, 24.5667°S, 46.8333°E, 800 m, rainforest, 17.xi.1992 (*B.L.Fisher*).

### Prionopelta xerosilva

Taxon classificationPlantaeHymenopteraFormicidae

Overson & Fisher sp. n.

http://zoobank.org/794FF570-F328-49F0-A5D7-44775B31A230

**Figure 12.**
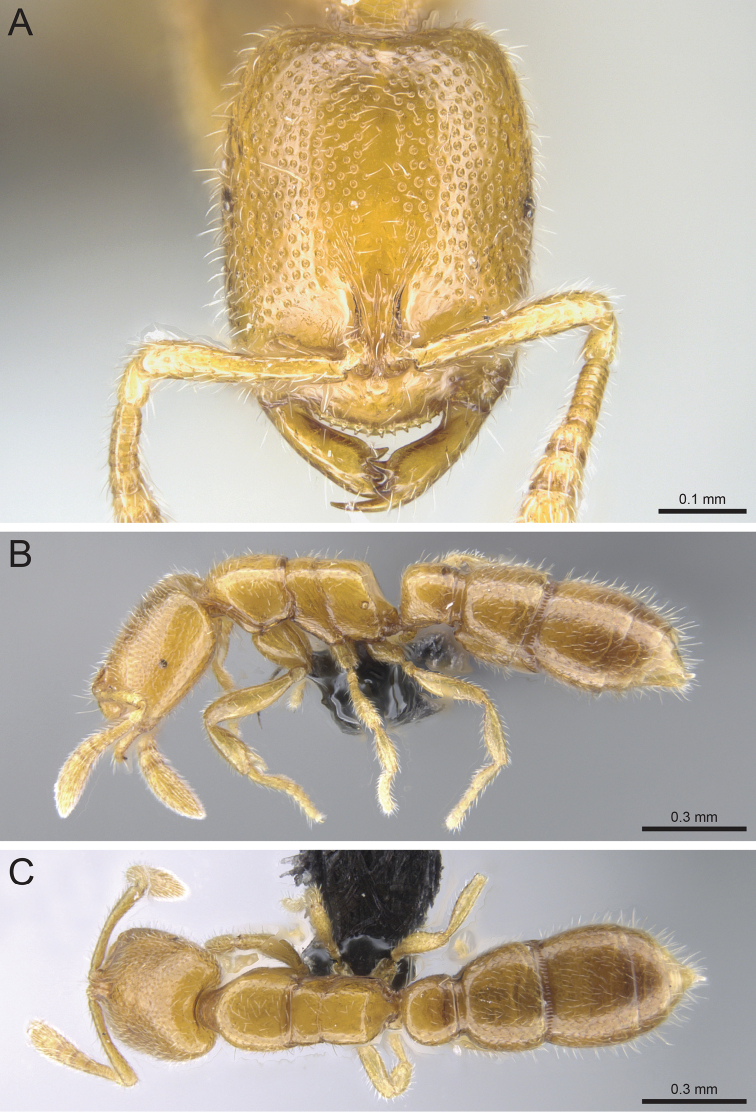
*Prionopelta xerosilva* holotype worker (CASENT0157254). **A** Head in full-face view **B** Body in profile **C** Body in dorsal view.

Figs 1A , 2B, D , 12

#### Type material.

**Holotype**, pinned worker, MADAGASCAR, Mahajanga, Réserve Forestière Beanka, 50.7 km E Maintirano, 17.88021°S, 44.46877°E, 140 m, tropical dry forest on tsingy, sifted litter (leaf mold, rotten wood), BLF22999, 29.x.2009 (*B.L. Fisher et al.*) (CASC: CASENT0157254). **Paratypes**, six pinned workers with same data as holotype (CASC: CASENT0157257; CASENT0157258; CASENT0157260; CASENT0157262; CASENT0157701, CASENT0157702).

#### Diagnosis.

*Prionopelta xerosilva* is identifiable from the following combination of characters: cephalic foveae consistently and widely separated by 1–3 foveal diameters, with foveae directly adjacent only extremely rarely, including at the posterolateral corners of the head; metanotal suture strongly visible in dorsal view.

#### Worker measurements

**(N=6).** HL 0.43–0.46 (0.45); HW 0.34–0.36 (0.35); SL 0.23–0.25 (0.24); WL 0.45–0.5 (0.48); PetL 0.14–0.15 (0.15); PetW 0.19–0.21 (0.2); T1W 0.31–0.33 (0.32); CI 74.35–81.82 (77.85); PI 126.8–148.18 (138.76); SI 65.51–71.88 (69.77).

#### Worker description.

Cephalic foveae shallow, large, and most widely spaced of the Malagasy *Prionopelta*; directly adjacent cephalic foveae either completely lacking, or very rare; if any foveae are adjacent, then always with only 2–3 foveae connected, and these usually located medially on the head in full-face view; majority of cephalic foveae are separated by 1–3 foveal diameters, appear cleanly scooped from shining integument, and lack any raised margins at their perimeter; median cephalic band devoid of foveae is wide along its length, not tapering posteriorly; coronal suture absent medially on the head; apical tooth intermediate in length, never more than 4 times as long as the third tooth from base to tip (Fig. 2D); very weak, shallow, foveae present on the pronotum which are noticeably less dense than those of other Malagasy *Prionopelta*; foveae of the dorsum of the mesosoma interspersed with punctures; pronounced metanotal suture visible in dorsal view, mesopropodeal suture weaker but present; posterior edge of the propodeum straight or only very slightly concave in dorsal view; protruding lamellae of the posterior propodeum absent.

#### Etymology.

The name of this new species is a combination of the Greek adjective “xero” meaning dry and the Latin noun “silva” for forest, as this species is known only from tropical dry forests in western Madagascar.

#### Distribution and ecology.

**Figure 13.**
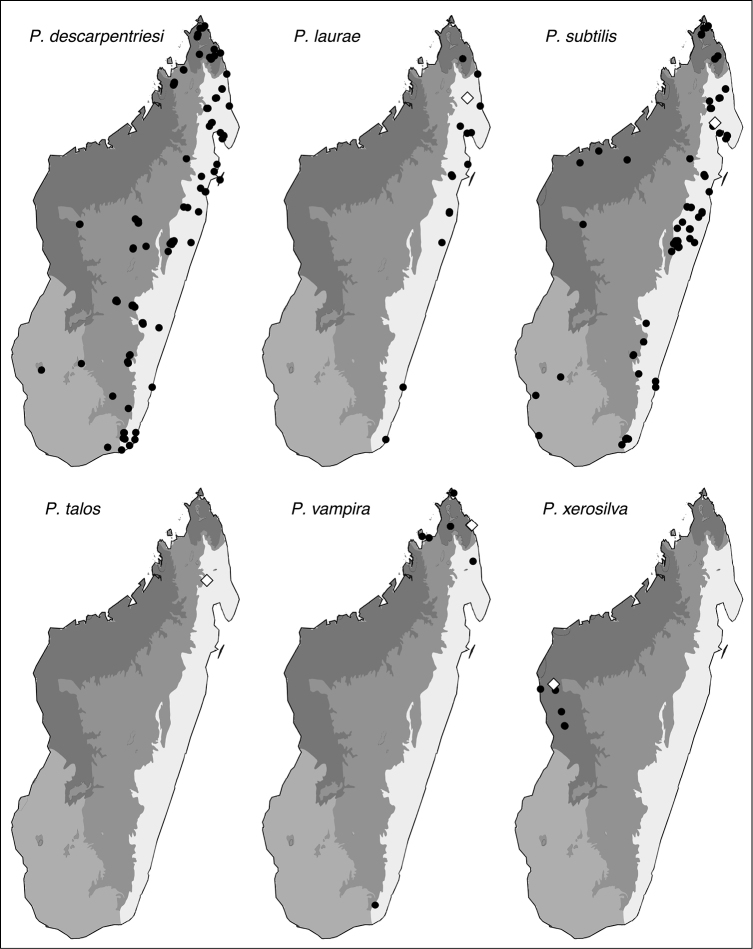
Geographic distributions for Malagasy *Prionopelta*. White diamonds represent type localities whereas dark circles represent non-type localities. Type locality for *Prionopelta descarpentriesi* is unknown. *Prionopelta seychelles*, known only from Seychelles, is not pictured here.

*Prionopelta xerosilva* is known only from tropical dry forests in the province of Mahajanga in western-central Madagascar (Fig. 13). It has only been collected from forest litter and ranges between 50–300 meters of elevation.

#### Taxonomic notes.

With its uniformly and widely spaced cephalic foveae, *Prionopelta xerosilva* could only be confused with *Prionopelta vampira*. However, the latter lacks a metanotal suture and possesses a very long apical tooth, a strongly concave posterior edge of the propodeum in dorsal view, and lamellae protruding from the posterior propodeum, all of which *Prionopelta xerosilva* lacks.

**Figure 14.**
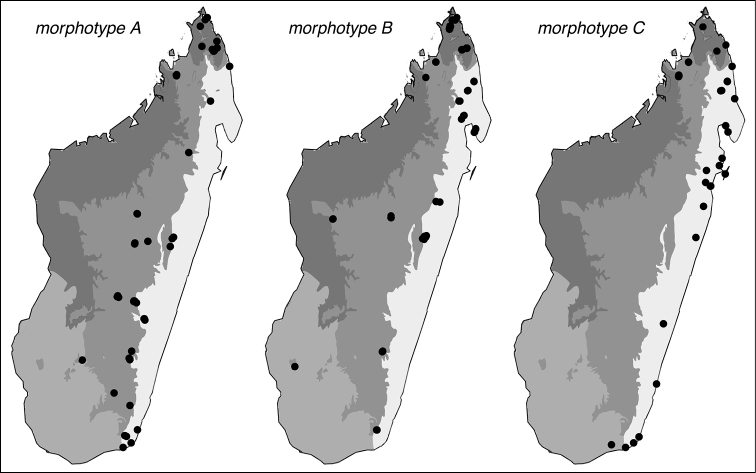
Geographic distributions for morphotypes of *Prionopelta descarpentriesi*.

#### Non-type material.

MADAGASCAR: Mahajanga, Parc National Tsingy de Bemaraha, 10.6 km ESE 123° Antsalova, 18.7094°S, 44.7182°E, 150 m, tropical dry forest on Tsingy, 16.xi.2001 (*Fisher-Griswold Arthropod Team*); Mahajanga, Parc National Tsingy de Bemaraha, 2.5 km 62° ENE Bekopaka, Ankidrodroa River, 19.1322°S, 44.8147°E, 100 m, tropical dry forest on Tsingy, 11.xi.2001 (*Fisher-Griswold Arthropod Team*); Mahajanga, Parc National Tsingy de Bemaraha, 3.4 km 93° E Bekopaka, Tombeau Vazimba, 19.1419°S, 44.828°E, 50 m, tropical dry forest, 6.xi.2001 (*Fisher-Griswold Arthropod Team*); Mahajanga, Réserve forestière Beanka, 50.2 km E Maintirano, 18.0265°S, 44.0505°E, 250 m, tropical dry forest on tsingy, 19.x.2009 (*B.L.Fisher et al.*); Mahajanga, Réserve forestière Beanka, 52.7 km E Maintirano, 18.0622°S, 44.5259°E, 300 m, tropical dry forest on tsingy, 24.x.2009 (*B.L.Fisher et al.*).

## Supplementary Material

XML Treatment for Prionopelta descarpentriesi

XML Treatment for Prionopelta laurae

XML Treatment for Prionopelta seychelles

XML Treatment for Prionopelta subtilis

XML Treatment for Prionopelta talos

XML Treatment for Prionopelta vampira

XML Treatment for Prionopelta xerosilva
